# Integrating Intracellular Dynamics Using CompuCell3D and Bionetsolver: Applications to Multiscale Modelling of Cancer Cell Growth and Invasion

**DOI:** 10.1371/journal.pone.0033726

**Published:** 2012-03-26

**Authors:** Vivi Andasari, Ryan T. Roper, Maciej H. Swat, Mark A. J. Chaplain

**Affiliations:** 1 Division of Mathematics, University of Dundee, Dundee, Scotland, United Kingdom; 2 Issaquah, Washington, United States of America; 3 Biocomplexity Institute and Department of Physics, Indiana University, Bloomington, Indiana, United States of Americs; University of Connecticut Health Center, United States of America

## Abstract

In this paper we present a multiscale, individual-based simulation environment that integrates CompuCell3D for lattice-based modelling on the cellular level and Bionetsolver for intracellular modelling. CompuCell3D or CC3D provides an implementation of the lattice-based Cellular Potts Model or CPM (also known as the Glazier-Graner-Hogeweg or GGH model) and a Monte Carlo method based on the metropolis algorithm for system evolution. The integration of CC3D for cellular systems with Bionetsolver for subcellular systems enables us to develop a multiscale mathematical model and to study the evolution of cell behaviour due to the dynamics inside of the cells, capturing aspects of cell behaviour and interaction that is not possible using continuum approaches. We then apply this multiscale modelling technique to a model of cancer growth and invasion, based on a previously published model of Ramis-Conde et al. (2008) where individual cell behaviour is driven by a molecular network describing the dynamics of E-cadherin and 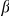-catenin. In this model, which we refer to as the centre-based model, an alternative individual-based modelling technique was used, namely, a lattice-free approach. In many respects, the GGH or CPM methodology and the approach of the centre-based model have the same overall goal, that is to mimic behaviours and interactions of biological cells. Although the mathematical foundations and computational implementations of the two approaches are very different, the results of the presented simulations are compatible with each other, suggesting that by using individual-based approaches we can formulate a natural way of describing complex multi-cell, multiscale models. The ability to easily reproduce results of one modelling approach using an alternative approach is also essential from a model cross-validation standpoint and also helps to identify any modelling artefacts specific to a given computational approach.

## Introduction

### 0.1 About Multiscale Modelling

Computational models of complex biomedical phenomena, such as tumour development, are becoming an integral part of building our understanding of underlying cancer biology. Mathematical models which are generated from biological data and experiments, *e.g.*, *in vivo* or *in vitro*, through phenomenological observations in real patients help in explaining the mechanisms of this complex phenomenon. Quantitative, predictive models have the potential to significantly improve biomedical research by allowing virtual, *in silico* modelling.

Experimentalists and theoreticians have agreed that cancer progression involves processes that interact with one another and occur at multiple temporal and spatial scales. The time scales involved vary from nanoseconds to years: signalling events in the cell typically occur over fractions of a second to a few seconds, transcriptional events may take hours, cell division and growth and tissue remodelling require days, tumour doubling times are on the order of months, and tumour growth occurs over years, etc. Typical spatial scales range from nanometres for protein-DNA interactions to centimetres for a the development of a solid tumour mass, tumour-induced angiogenesis, tissue invasion, etc. These scales are strongly linked with each other. A phenomenon cannot be completely considered using a single scale, fully isolated without taking into account what happens at other smaller or larger scales.

In general, when incorporating different temporal and spatial scales into mathematical models, there are three commonly used viewpoints: the subcellular level, the cellular level, and the tissue level. Or, from a modelling point of view these levels can also be referred to as the microscopic scale, the mesoscopic scale, and the macroscopic scale, respectively. Cancer usually starts at the subcellular level marked by events that occur within the cell, such as genetic mutations, transduction of chemical signals between proteins, and a large number of intracellular components that regulates outward activities at the cellular level such as uncontrolled cell division, and cell detachment that leads to epithelial-mesenchymal transition (EMT), etc. The main activities of cell populations, such as interactions between tumour cells and host cells, intravasation and extravasation processes, proliferation, apoptosis, aggregation and disaggregation properties, are all viewed from a larger scale, that is the mesoscopic scale. The macroscopic scale concerns activities that occur at the tissue level such as cell migration, convection and diffusion of chemical factors, all of which are typical for continuum processes [1].

During the last decade or so many approaches to multi-cell, multiscale modelling of cancer growth and treatment therapy have been developed. For example, see articles by [2]–[20] for modelling details and [21]–[23] for reviews on multiscale modelling. The goal of each approach is, in the first instance, to be able to replicate observed experimental results and data. Since the biology of cancer is very complex, models have to focus on “first order” effects and introduce certain simplifications to make them computationally feasible. These simplifications often introduce modelling artefacts *i.e.*, observed model behaviours or side effects which are due to the particular choice of the mathematical/computational method. Isolating the source of modelling artefacts is very difficult and quantifying the impact of such modelling artefacts on model predictions is a daunting task. Therefore, in order to identify deficiencies and limitations of modelling methods currently in use, we have to be able to routinely conduct rigorous model cross-validation to ensure that predictions of different modelling approaches for a single biological system are in agreement, at least qualitatively, with each other and with experimental data. Since in many situations experimental data is hard to find or simply unavailable model, the issue of model cross-validation is even a more important issue.

For mathematical models of biomedical systems to be credible and usable on a larger scale by a variety of biomedical researchers, they have to be: a) easy to set up, b) easily reproducible, c) transparent and open to peer review and challenge, d) publicly accessible and able to run on multiple operating systems without the need to recompile, and e) interactive and easily modifiable.

In this paper we present a case study on model cross-validation. We reproduce a cancer invasion model, originally described in [14], using a CC3D-based implementation and compare our simulation results to those of the original paper (in which a centre-based implementation was used). We document the details of model building based on the published article, highlight obstacles in reproducing published results and suggest a streamlined, systematic approach to cell-based model cross-validation.

### 0.2 CC3D-Bionetsolver framework for multiscale simulation

Modelling methodologies that explicitly represent individual cells are particularly appropriate for modelling and simulation of cancer invasion. There are important events and physical phenomena associated with cancer invasion on the single-cell level that can only be suitably captured in computational simulations by accounting for individual cell properties and important aspects of cell-cell interactions, such as changes in cell-cell contact area.

In modelling the various stages of cancer progression, certain computational and mathematical methodologies are more suitable than others. For example, in the case of solid avascular tumour growth, continuum models are well-suited since they capture bulk properties of tissues. Instead of explicitly treating individual cells, collective properties of the whole tumour tissue are modelled, such as cell density and oxygen concentration. An advantage of such an approach is that systems with a large number of cells, such as on the order of 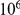 or higher, can be handled. On the other hand, explicit representation of individual cells and their properties (*e.g.*, locations, radii, morphology, surface area, volume, etc.) can become computationally burdensome when trying to model on the order of 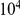 to 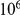 cells. Nevertheless, such individual cell-based modelling approaches are capable of capturing phenomena and behaviour in multicellular systems that continuum strategies cannot capture.

Systematic development of biomedical models may be divided into the following distinct stages: a) creating a conceptual biomedical model, b) developing a formal description of the model based on an established modelling language such as the Systems Biology Markup Language or SBML, c) translating the formal language into a set of mathematical representations, for example, SBML is translated into a set of ordinary differential equations or ODEs, and d) developing a computational implementation of c).

“Traditional” biomedical model building usually skips intermediate stages and jumps from a conceptual model description directly into low-level code. This is often convenient from the perspective of a modeller but it greatly impedes model cross-validation, reuse or sharing. Problem solving environments, such as CC3D, Mason, or Flame, greatly reduce the amount of effort necessary to build models which rigorously follow stages a)–d) and at the same time offer the same level of flexibility in model construction as low-level programming languages. To build and run our models we used CC3D - an open source simulation environment based on the Glazier-Graner-Hogeweg (GGH) model which allows simulating cell behaviours on an individual cell basis, where individual cells can interact with each other or with the underlying medium. Several models of tumour growth and angiogenesis have already been simulated using CC3D environment. See, for example, articles by [24]–[28].

Multiscale models in CC3D-Bionetsolver are described using a combination of the CompuCell3D Markup Language (CC3DML) and Python scripting. Such a combined approach allows one to build complex biomedical models and does not require recompilation when running them. In a typical CC3D simulation “static” aspects of the model, such as lattice size, simulation runtime, list of cell types, initial conditions or cadherin affinities, are usually described using CC3DML. We can replace CC3DML with equivalent Python syntax. The “dynamic” part of the CC3D model is described using Python scripting. Since Python is a full-featured programming language, modellers are able to express complex cell type differentiation rules, couple cell properties to concentrations of diffusive chemicals or to cell-cell signalling or parameterise cell adhesive properties in terms of underlying molecular or gene regulatory networks.

### 0.3 Comparison of center-model and GGH-model for multicellular simulation

**Figure 1:**
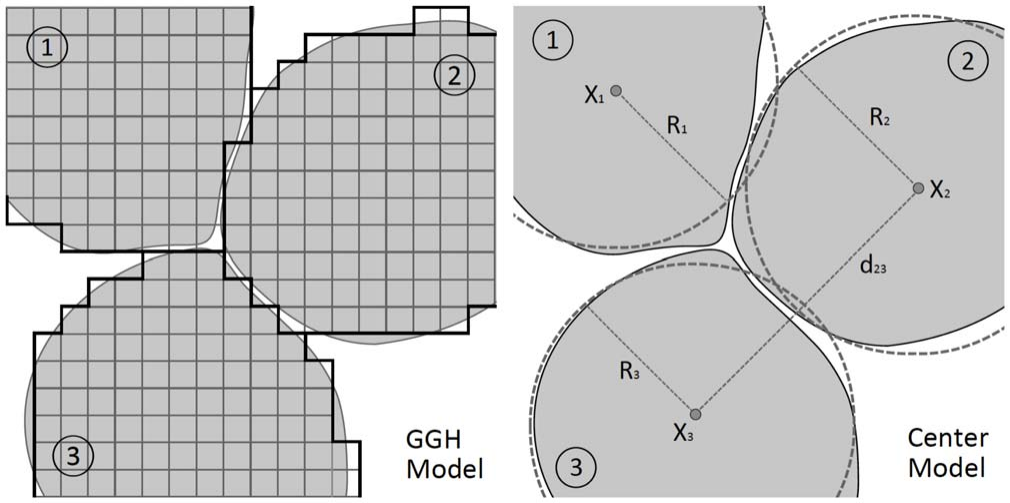
Schematic illustration of a lattice-based representation of cells in the GGH model (left figure) and a lattice-free representation in the centre-based model (right figure).

Here we briefly discuss the main differences and some similarities between the centre-based model of [14] and our model based on the GGH model. As indicated, CC3D is a software application that implements the GGH model, allowing lattice-based simulation of multicellular systems. Each biological cell is represented as a set of contiguous sites on a lattice and the system evolves in time through an energy minimization procedure. On the other hand, the centre-based model represents each biological cell in terms of the location of its centre of mass and its radius. This fundamental distinction between the two methodologies is illustrated in Fig. 1.

The cells of the centre-based model behave as elastic spheres and equations describing their behaviour and interactions are derived on the basis of classical mechanical concepts. The centre-based model approximates cell-cell contact areas using the radii of neighbouring cells and the distance between their centres. In contrast, the concept of cell neighbour has an explicit representation in the GGH model since two cells share one or more lattice edges (for 2D simulations) or faces (3D simulations). Because of these differences, each modelling approach has relative strengths and weaknesses with respect to capturing different biophysical processes and phenomena. On the other hand, the GGH and centre-based models also have some important similarities. Both methodologies use continuum, reaction-diffusion equations to model extracellular chemical fields and they both incorporate cell-cell adhesion and mechanical constraints on cell shape. In each case, extracellular chemical fields can both modify and be modified by cell behaviours or properties such as cell growth rates, secretion, absorption and chemotaxis.

### 0.4 An application to multiscale modelling of cancer growth and invasion

The multiscale model of epithelial-mesenchymal transition (EMT) developed by [14] incorporates important aspects of E-cadherin-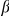-catenin signaling and its coupling to cell-level properties of intercellular contact and adhesion. This model requires explicit representation (on a cell-to-cell basis) of localised and spatially heterogeneous changes in cell-cell adhesion strength and contact areas. It is at this level of granularity that invasive cancer cells sense and respond to their environment. In terms of biological processes, the model of [14] captures cell-contact-dependent recruitment of E-cadherin and 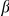-catenin to the cell membrane and reincorporation of both back into the cytoplasm. Computationally, the simulations incorporated (1) time-varying changes in cell-cell adhesion as a function of a system of ordinary differential equations (ODEs) for intracellular reaction kinetics of E-cadherin-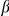-catenin signalling and (2) changes in rate parameter values in the reaction kinetic model as a function of changing contact areas between neighbouring cells.

## Results

We ran three sets of 3D simulations to model: (1) detachment waves of 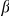-catenin in a thin layer of epithelial cells, described in subsection 0.5, (2) tumour growth and detachment of cells from a layer of epithelial cells, and (3) tumour growth and detachment of cells from a multicellular tumour spheroid, both described in subsection 0.6.

Initially, all cells were individually created in the shape of a cube of size 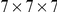 pixels, with gaps of 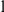 pixel length between them. From 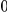 MCS to 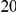 MCS we allow the cells to grow, during which time the volumes and surface areas of the cells increase and the cells become more spherical. During this period of the simulations, cell-cell contact areas undergo an equilibrating transient that does not reflect natural phenomena. Thus, we did not start the numerical integration of the differential equations (corresponding to the subcellular biochemical networks) until 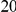 MCS. Keeping in mind that the subcellular model is sensitive to changes in intercellular contact areas, if numerical integration occurred during the initial cell shape changes, unrealistic subcellular dynamics could occur as an artefact of these changes. Starting the integration at 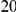 MCS helped avoid this. All parameter values used in the computational simulations are listed in Table 1, unless stated otherwise.

**Table 1:** Dimensionless intracellular parameter values for the cell detachment simulations.

Parameter	Definition	Value	Reference
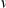	E-cadherin- 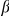 -catenin binding rate	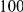	[14]
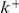	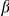 -catenin-proteasome downregulated binding rate	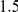	Estimated
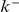	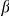 -catenin-proteasome dissociation rate	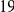	[14]
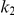	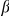 -catenin degradation rate in proteasome	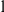	[14] *
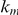	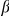 -catenin production rate	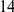	[14] *
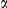	E-cadherin- 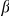 -catenin dissociation rate	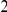	[14]
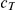	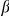 -catenin threshold value	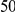	[14]
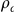	E-cadherin cytoplasm-surface translocation rate	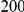	[14]
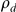	E-cadherin surface-cytoplasm translocation rate	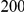	[14]
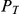	Proteasome total concentration	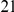	[14] *
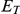	E-cadherin total concentration	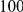	[14]

* appears in the paper's correction.

### 0.5 Detachment Waves of Epithelial Layer Simulations

**Figure 2:**
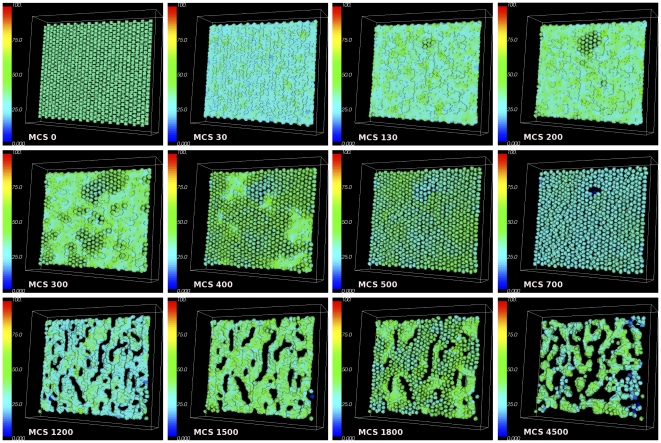
Plots showing a sequence of the disruption of a layer of epithelial cells due to an increase in the 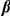-catenin concentration inside the cells. After all cells have detached from the layer of cells or from each other (EMT), 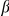-catenin concentrations eventually drop, causing cells that are close to each other to undergo re-attachment (MET) while other cells that are not close remain as mesenchymal cells. Colours of the cells correspond to concentration of 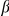-catenin.

To simulate detachment waves of 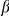-catenin in a thin layer of epithelial cells, we performed the simulation on a domain or a lattice of 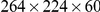 pixels in 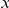, 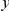, and 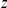 directions, respectively, with the 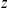-axis being perpendicular to the page. In the lattice, we place a sheet of cells with 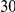 cells along the 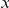-axis (horizontal), 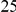 cells along the 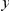-axis (vertical), and 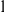 cell along the 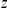-axis. As mentioned, initially each cell occupies a cube 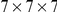 pixels and we insert a gap of 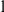 pixel between each cell, as can be seen in the top left figure of Fig. 2. The aim of giving a 1-pixel gap for this simulation is to give space for the cells to grow where cell volume increases followed by increasing cell surface area until the cells become spherical and tightly attached to each other, as can be seen from the top second left figure (MCS 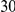) of Fig. 2. The initial target volume for cells is set to 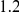 times the cell volume, making the average volume of each cell about 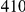 pixels. We set 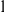 pixel equal to 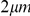. Therefore one tumour cell has a volume of about 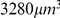. The sheet represents a thin layer of tissue with a volume of 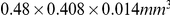.

In the intracellular model, summarised in Eqs. (15)–(18), disruption of cell-cell adhesion occurs when there is an increase in the concentration of free 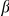-catenin in the cytoplasm, that is when 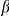-catenin concentration exceeds a specified threshold value as a result of disassociation of E-cadherin-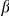-catenin complex at the cell membrane. The threshold we specified for our simulations is 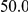. As explained previously, for cell detachment to occur, nuclear 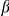-catenin must exceed this threshold value.

Depending upon whether 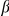-catenin is above or below the critical EMT-MET threshold, the terms 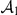 and 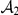 in Eqs. (11) and (12) are changed appropriately. In each case, if 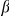-catenin is below the threshold, the first terms in Eqs. (11) and (12) are used. On the other hand, if 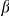-catenin is above the threshold, the second terms in each of the equations are used. However, in the SBML implementation of our subcellular model, we do not actually implement two separate sets of equations for attached (below-threshold) and detached (above-threshold) cells. Instead, we include both terms from Eq. (11) and both terms from Eq. (12) in the same SBML file. We effectively “include” or “omit” one term or the other (depending on whether cells are below-threshold or above-threshold) by either (1) setting 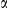 equal to 0 and 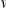 equal to a non-zero value (see 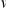 in Table 1) for the case of a below-threshold cell or (2) setting 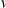 equal to 0 and 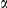 equal to a non-zero value (see 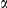 in Table 1) for the case of an above-threshold cell.

In our CC3D-Bionetsolver implementation (*i.e.*, our Python script), the increase of 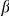-catenin concentration above threshold is deliberately initiated by decreasing the value of 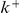 at a specified time (70 MCS) from 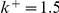 to 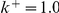. This parameter influences the association rate of 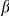-catenin with the proteasome. When 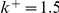, 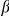-catenin-proteasome complex formation is sufficiently rapid to keep the 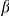-catenin concentration of all cells well below the threshold of 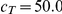. However, when 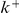 is decreased to a value of 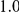, 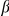-catenin accumulates in the cytoplasm as a result of decreased proteasomal degradation.

We check the 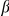-catenin concentration for every cell at each MCS. If the 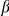-catenin concentration for a cell of type “LowBetaCat” increases above a threshold value of 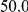, the cell type is changed to “HighBetaCat”, 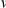 is set to 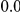 instead of 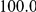 and 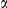 is set to 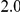 instead of 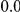. Similarly, when the 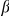-catenin concentration of a “HighBetaCat” cell decreases below the threshold, the value of 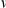 for that cell is set to 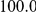 and 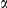 is set to 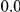 and the cell type is switched to “LowBetaCat”.

In the case of an EMT event (*i.e.*, a cell type change from “LowBetaCat” to “HighBetaCat”), changing the values of 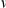 and 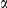 as described is equivalent to swapping the expressions in 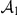 and 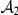, between the below-threshold (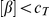) expressions and the above-threshold (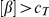) expressions. Physically, this corresponds to (1) a cessation of E-cadherin-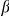-catenin complex formation in the membrane (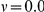) and (2) an accelerated dissociation of E-cadherin-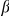-catenin complex (*i.e.*, the dissociation rate parameter 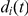, is increased by 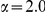) to form cytoplasmic (free) E-cadherin and free 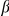-catenin. Together, the effects of these two phenomena are (1) an increased concentration of 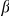-catenin in the cytoplasm and (2) a significantly reduced adhesion strength between the transformed cell and its neighbouring cells due to the loss of E-cadherin-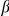-catenin complex in the membrane.

In Fig. 2 an increase of 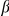-catenin above threshold occurs in several cells, randomly. When one cell is induced with a high 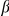-catenin concentration above the threshold 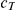, the cell becomes vulnerable to a loss of cell-cell attachment resulting in EMT. The event propagates outward from this localised event, affecting neighbouring cells. When a given cell detaches, the neighbouring cells in turn become vulnerable to EMT because of increased free 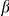-catenin concentration inside the neighbouring cells. These cells detach from surrounding cells and the effects propagate throughout the layer of cells. At 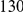 MCS, we observe a small group of cells that start to detach. By 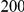 MCS, detachment waves have spread outward to adjacent cells. As time evolves, some cells at other positions also show detachment waves independently. Eventually around 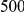 MCS all cells in the layer have been affected and have detached from each other. This is the hallmark of EMT events. Due to the stochastic nature of the GGH model, regular waves of cell detachment which originate from one cell and then spread radially and regularly outward as seen in [14] can not be produced using CompuCell3D. Nevertheless, the results are qualitatively the same between our implementation and that of [14]. Also, our aim in this paper is to illustrate differences between the two approaches. They are different in different ways and we may biologically conjecture that neither is superior to the other.

**Figure 3:**
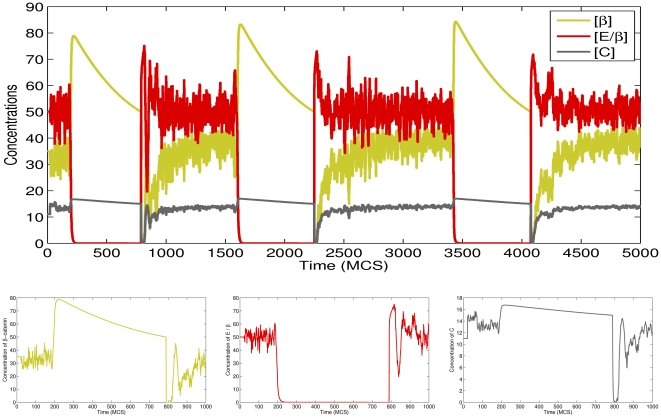
Plots of 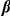-catenin, E-cadherin-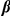-catenin complex, and proteasome-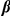-catenin concentrations for a simulation in which cells undergo epithelial-mesenchymal transition (EMT) and subsequently recover by mesenchymal-epithelial transition (MET). The cells reattach to adjacent cells and thereby reform an epithelial layer. The cycle of detachment and reattachment occurs about 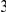 times until 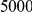 MCS.

In order to see how the concentrations of proteins inside individual cells vary over time, we wrote functions in our CC3D-Bionetsolver code to record values to output files of all concentrations each MCS. In Fig. 3, we plot concentrations of 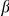-catenin, E-cadherin-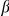-catenin complex, and 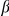-catenin-proteasome complex for a typical cell undergoing EMT and MET (see the simulation results shown in Fig. 2). Because of the stochastic nature of the GGH model, the concentrations fluctuate in response to fluctuations in contact area between cells. When the concentration of 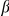-catenin increases significantly (due to loss of contact area between cells and complete detachment) the transition curve (during the period of detachment) becomes smooth (*i.e.*, fluctuations cease). After the cell regains contact with other cells, the curve is observed to fluctuate again. The top figure of Fig. 3 shows the concentrations of 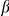-catenin, E-cadherin-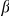-catenin complex, and 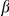-catenin-proteasome complex when running the simulation up to 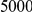 MCS. Here we see three cycles of detachment and attachment, as shown from the repeated cycles of high and low concentrations of 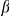-catenin (yellowish-green line).

Once the cells have detached from each other, they are free to randomly migrate from their original positions. However, in this simulation we did not apply any source of attractants that should cause the cells to migrate away from the layer; the cells detach but stay in their position or slightly move due to the stochastic nature of CC3D. Allowing the simulation to proceed all the way to 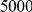 MCS, we observe that 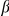-catenin concentrations in detached cells gradually decrease back toward the threshold. When the concentration reaches the threshold, 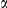 in the internal model is again set to zero and 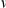 is set to a non-zero value. This alters the internal kinetics such that 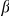-catenin is no longer rapidly degraded. Instead, it accumulates inside the cells and is reincorporated into E-cadherin-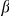-catenin complex. This process occurs rapidly so that near-zero concentrations of free 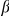-catenin are observed in some cells as seen in the bottom left figure of Fig. 3 (bottom figures are plots of the concentrations to 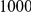 MCS or for one cycle of detachment).

The increase of E-cadherin-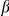-catenin complex increases the adhesiveness of cells and they undergo mesenchymal-epithelial transition (MET) resulting in the reattachment of neighbouring cells. Thus, cells again exhibit an epithelial phenotype, but this time with an irregular configuration of the cell layer, or loss of epithelial configuration. This is because of the random migration of cells away from their original positions that occurred when they were detached. The results we report here resemble those in Fig. 7 of [14]. We also observe from the simulation results that, after the first stage of EMT events, a few cells migrate so far that they cannot reattach to other cells. These cells remain as mesenchymal cells.

**Figure 4:**
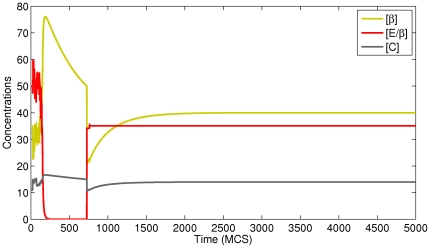
Plots of 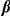-catenin, E-cadherin-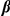-catenin complex, and proteasome-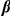-catenin concentrations for a typical cell undergoing epithelial-mesenchymal transition.

As for cells that cannot reattach after the first detachment (because they have migrated too far from other cells and thus remain mesenchymal), the concentrations of the subcellular proteins immediately reach their own steady states, as shown by plots of data in Fig. 4.

### 0.6 Tumour Growth and Invasion

For simulations involving tumour growth, the GGH target volume is incremented each MCS during growth phases at a constant rate of 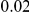 times the current cell volume and GGH target surface area is also incremented at a constant rate of 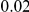 times the current cell surface area. This results in a doubling of cell number approximately every 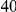 MCS. Cell division was set to occur when the volume of a cell exceeded 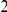 times its initial volume. This rate of growth was not necessarily intended to reflect *in vivo* rates of tumour cell growth. Rather, the purpose in our simulations is simply to let the tumour grow to a specified size so that we can then initiate EMT events and observe the subsequent dynamics of cell detachment and migration.

#### 0.6.1 Tumour from a Layer of Cells

**Figure 5:**
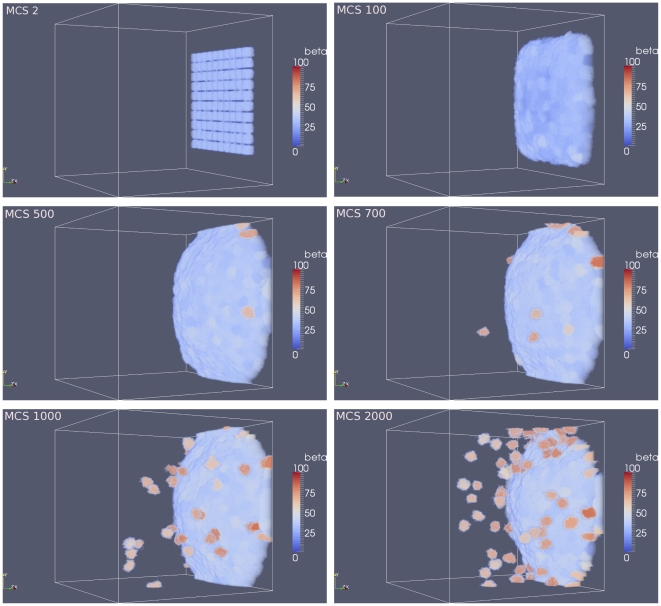
Plots showing the results of a simulation of tumour growth and local invasion (detachment) from a layer of cells. The tumour grows rapidly from a single layer and eventually EMT events are observed to occur. Cell colour represents 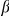-catenin concentration.

To simulate the growth of a tumour from a layer of cells (common for tumours of epithelial tissue origin) we use a larger 3-dimensional lattice or a cubic lattice of size 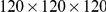 pixels in 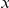, 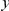, and 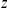 directions. Initially we place one layer of cells (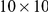 cells) at one face/side of the cube (at 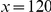) as seen in the top left figure in Fig. 5. All cells start out cube-shaped with size 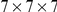 pixels and a 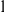 pixel gap between each of them. In this simulation, we apply a linear concentration gradient of chemoattractant in the 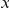-axis direction to generate cell migration.

From a single thin layer, the tumour grows and becomes a bulky layer as a result of rapid cell division. In the implementation of CC3D-Bionetsolver, it is possible to let the tumour grow indefinitely, but in this simulation we limit the cell division. Cells are permitted to grow and divide until the total number of cells in the tumour mass exceeds 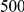 cells. After 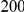 MCS we initiate an increase of free 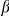-catenin concentration, as previously described, by reducing the value of 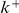 from 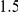 to 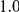. Beginning around 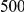 MCS, some cells in the outer layer show high concentrations of free 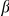-catenin. These cells eventually break away from the primary tumour mass and migrate in the direction of increasing chemoattractant concentration (away from the tumour mass).

As EMT events propagate over the tumour surface and more cells begin to detach from the outer layer, cells underneath the surface are exposed to the medium. The reduced amount of cell-cell contact area that these underlying cells experience destabilises them and makes them vulnerable to EMT. The 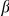-catenin concentrations in these cells increase above threshold and eventually the cells undergo EMT and detach from the tumour. In this way, the effects of early EMT events propagate into the tumour surface as the tumour mass grows and a continual series of detachment events are observed to occur.

**Figure 6:**
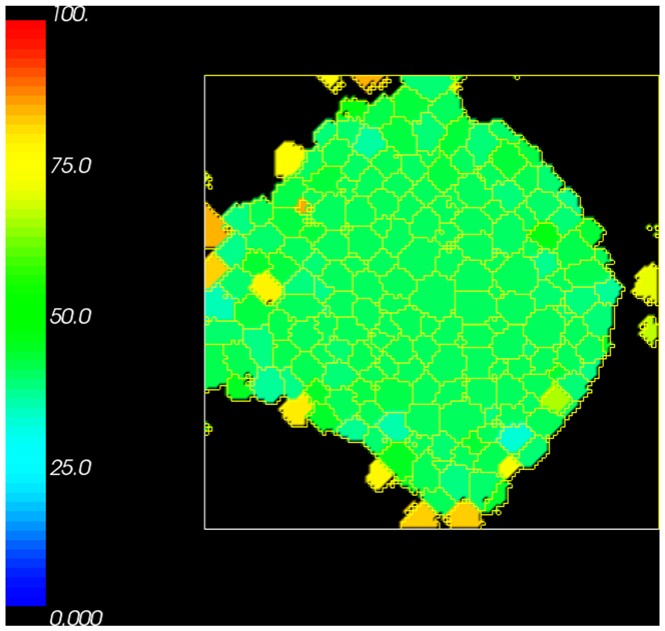
Plot of a cross sectional view showing the spatial distribution of 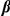-catenin concentration inside cells from the simulation of tumour growth from a layer of cells. Cells in the centre of the tumour mass have a large number of binding neighbours, hence the concentration of 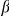-catenin is lower than the cells at the outer layer of tumour mass that have fewer binding neighbours and a high concentration of free 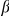-catenin.

To show the distribution of free 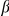-catenin inside the cells that remain within or attached to the primary tumour mass, we provide a cross sectional view of the tumour mass along the 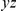 plane in Fig. 6. Cells that are bound to other cells inside the tumour are roughly blue in colour. This indicates a free 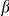-catenin concentration lower than the threshold 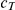. On the other hand, cells in the outer layer are exposed to medium and have less cell-cell contact area. The colour of these cells and those immediately underneath them range from yellowish green to dark orange. This indicates higher concentrations of free 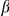-catenin near to or greater than 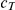. These results are in good agreement with the simulation results of [14] and experimental data of [29]. While our mathematical model does not explicitly model (or make a distinction between) the two types of 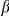-catenin, we assume that the concentration of free 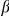-catenin inside the cytoplasm (which we explicitly model) provides some indication of the concentration of nuclear 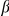-catenin.

To study the sensitivity of multiscale dynamics to the 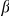-catenin degradation rate parameter 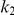, [14] performed simulations with different values of 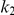 (corresponding to different degrees of tumour cell invasiveness in tumour invasion assays). The invasion assay has been used *in vitro* as a measure of invasive potential of tumour cells. In our simulations, if 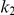 is small this results in high concentrations of free 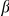-catenin. If concentrations exceed threshold, then cells are susceptible to cell-cell detachment and may become invasive by breaking away from the primary tumour mass. In other words, sufficiently small 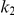 can be thought of as a marker for malignant or invasive tumour cells. [14] used 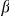-catenin degradation rate values of 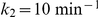 (fast degradation rate), 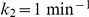 (medium degradation rate), and 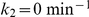 (no degradation).

**Figure 7:**
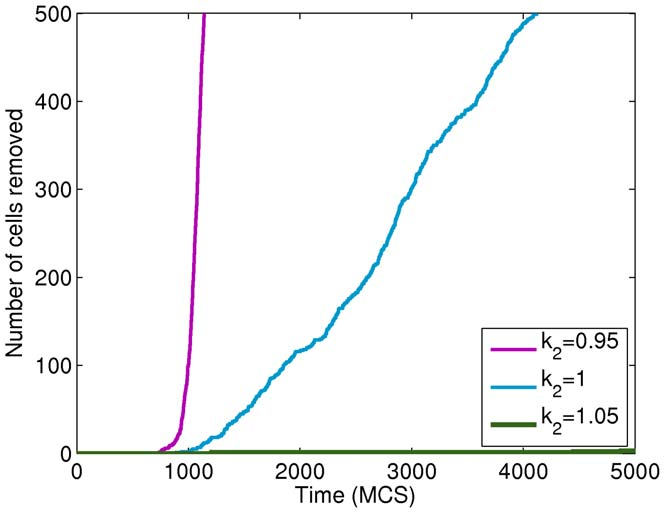
Plots showing the effect of varying the parameter 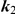 on the number of cells that detach from a primary tumour mass in a layer configuration. The value of 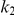 was varied between high, intermediate and low values and the number of cells that detach and migrate a certain distance from the tumour mass was monitored.

Our CC3D-Bionetsolver implementation is, for some reason, very sensitive to small changes in 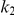. In other words, tumour cell invasiveness in our simulations varies significantly with only small variations in 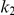 values (much smaller than those used in [14]). Because of this sensitivity, we only varied 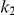 within a very small range using a value of 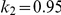 for the low degradation rate, 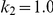 for the medium degradation rate and 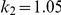 for the fast degradation rate. The resulting data that we collected from our simulations are summarised in Fig. 7, where, qualitatively, the results are the same as those in [14]. This illustrates the differences between the two approaches, which is the main aim of this paper. We have plotted the number of cells that reached a fixed distance over time. In the implementation, we remove cells that reach a certain distance from the main tumour mass. For this, we chose a distance 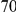 pixels. The maximum number of cells in the simulations (and therefore the maximum number of cells that can be removed) is 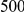 for all simulations. The curve obtained from the slow degradation rate simulation (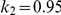) increases exponentially over a short period of time (purple line), while that obtained using 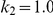 shows a more gradual increase in the number of removed cells (blue line). Finally, the curve corresponding to 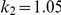 (the fast degradation rate invasion assay) increases very slowly, indicating that only a small number of cells detached and were removed beyond the distance threshold of 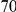 pixels (green line).

#### 0.6.2 Multicellular Spheroid Tumour (MTS)

It was also of interest to see how our CC3D-Bionetsolver implementation could mimic the growth and invasion of multicellular tumour spheroids or MTS. These are spherical aggregations of (malignant) cells that can be grown *in vitro*. MTS are particularly used in cancer research for studying multicellular resistance or chemo- or radiotherapy assays [30]. They can be used to study cell-cell and cell-matrix adhesion *in vitro* as well as the influence of the environment on many cellular functions including differentiation, cell death, apoptosis, gene expression and regulation of proliferation. MTS exhibit the characteristics of three-dimensional solid tumours.

**Figure 8:**
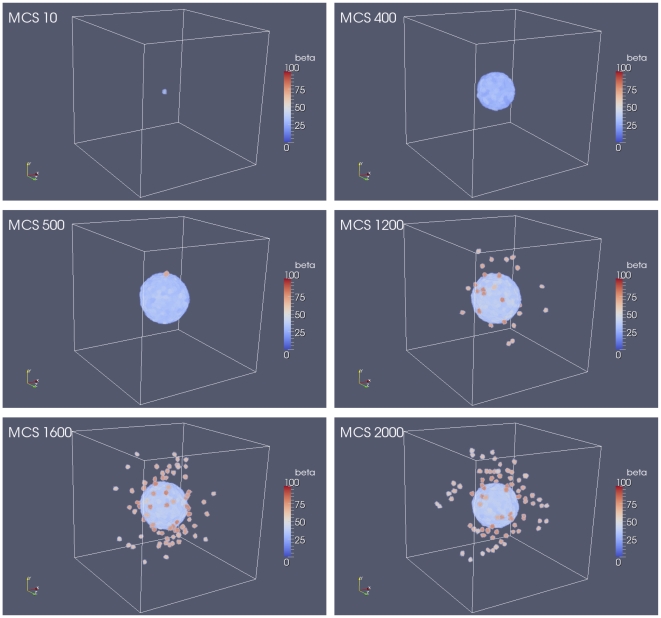
Plots showing the results of multicellular tumour spheroid simulations. The tumour grows from a single cell placed in the middle of a cubic lattice.

For MTS simulations, we use a cubic lattice with size 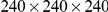 pixels in 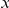, 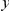, and 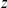 directions. The simulations begin with one cube-shaped cell (size 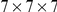 pixels) placed at the centre of the cubic lattice. To maintain tumour compactness as cells divide and to prevent undesirable effects before we trigger detachment, we set the threshold value of 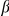-catenin (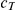) to a relatively high value (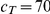). This ensures that no cells undergo EMT during the growth phase (in which the tumour is permitted to grow and become spherical in shape). An image of the tumour during this stage in the simulation is shown in the top right figure in Fig. 8. At 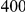 MCS the value of 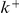 is decreased from 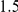 to 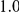 and at 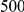 MCS cells at the surface of the tumour spheroid can be seen with a high concentration of free 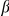-catenin. In these simulations, we apply a radial chemoattractant gradient increasing outwardly in all directions from a minimum value at the center of the cubic lattice. After losing cell-cell adhesion with neighbouring cells (due to EMT resulting from above-threshold concentrations of free 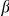-catenin), detached cells migrate radially outward in the direction of increasing chemoattractant concentration.

**Figure 9:**
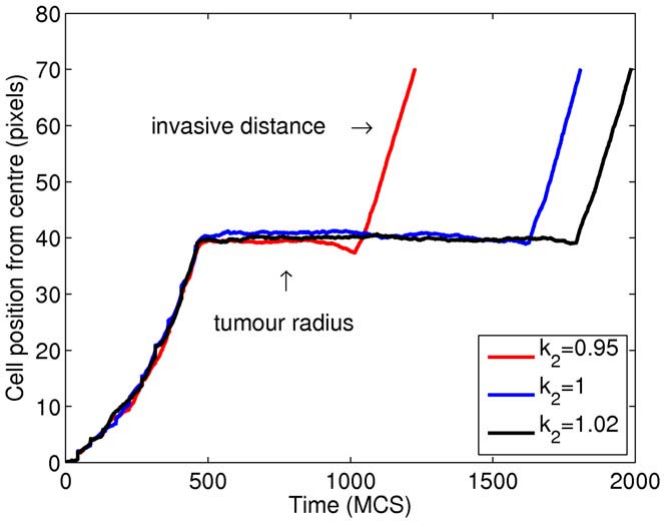
Position of cell ID 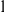 with respect to the centre of a cubic lattice of size 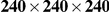 pixels during simulations of MTS using the following parameter values for 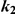: 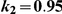, 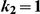, and 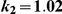. Tumour radius is apparent from the horizontal portion of the cell position time-courses. In each case (for all three parameter values) this occurs at a pixel value of 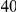.

An interesting feature of the data collected from MTS simulations is that it gives an indication of the size of the tumour. In Fig. 9 we show cell positions for a single cell over time in simulations with different values of 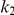. Cell positions with respect to an initial position (where the cell was created as a result of mitosis) were written to an output file for selected cells. All data in Fig. 9 were taken from cell ID 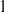, which actually was not created by mitosis, but instead was present in the initial lattice configuration at 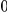 MCS. The data indicated by the red line were generated using a value of 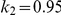, the blue line represents data using 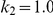, and the black line resulted from a simulation using 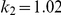. All data initially show an identical change in cell position from the centre of the lattice toward the same fixed position at 40 pixels. The cell resides here for an extended period of time before migrating quickly toward the edge of the lattice. This position of 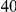 pixels can be assumed to be the radius of the MTS. All three simulations (using different values of 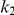) indicate the same value for tumour radius. On the other hand, for each 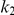 value, the cell detaches from the primary tumour mass at a different time. This can be seen in the latter portions of each of the curves. In each case, there is a portion of the time-course that increases linearly (indicating the cell has detached from the main tumour). This linearly increasing portion occurs at a different point in time for each of the three simulations.

In a study by [31] of the growth and invasion of glioblastoma multiforme (GBM) in 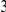-dimensional collagen I matrices, invasive distance is defined as the radius of the entire GBM system minus the radius of the MTS. Thus, in our simulations, invasive distance corresponds to the distance that cells move radially outward from the MTS after detaching from the primary tumour mass as seen in Fig. 9.

**Figure 10:**
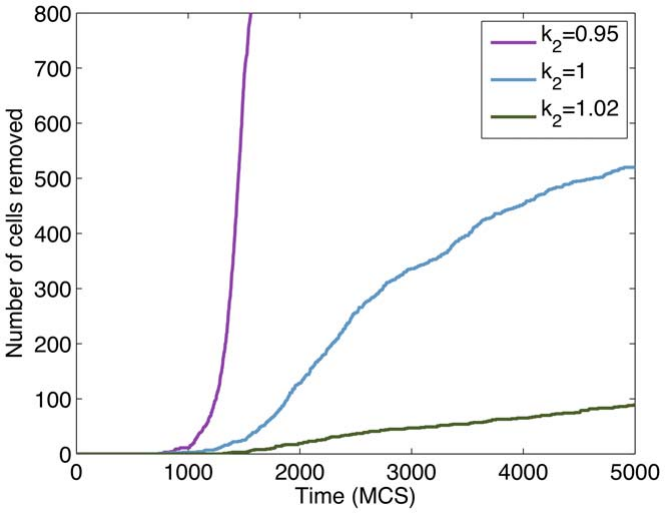
Plots showing the number of cells removed from MTS simulations using different values of 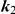 (corresponding to different levels of invasiveness).

Plots of invasion distance obtained from our MTS simulations show patterns similar to the data obtained from simulations using a layer of cells. This can be seen in Fig. 10. In the case of low 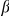-catenin degradation rate (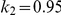), invasion assay data, indicated by the purple line, show an exponential increase in the number of cells that have reached a distance of 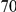 pixels (*i.e.*, have been removed from the simulations). For simulations using 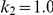 and 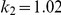, cell removal rates are slower than for the simulation using 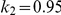, thus suggesting less invasive tumours.

**Figure 11:**
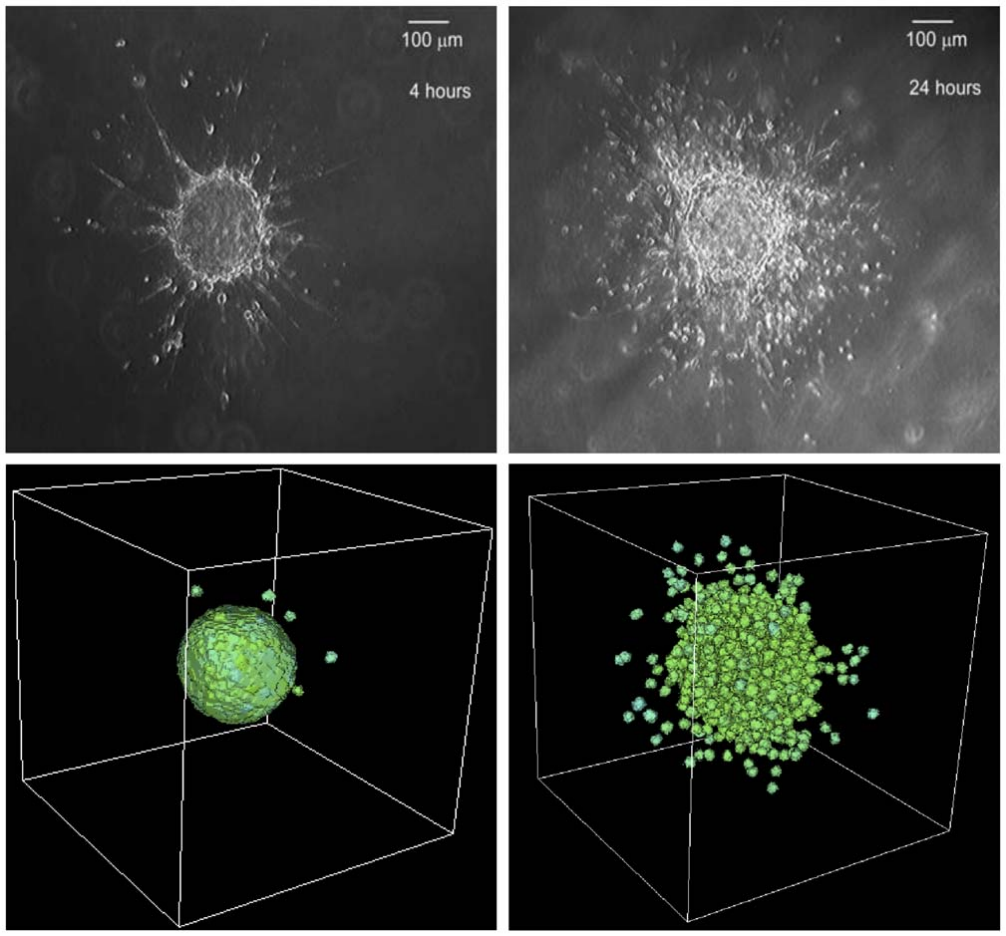
Comparison between our computational results with experimental data. Images showing experimental data of MTS growth patterns in low collagen concentration (top left figure), a less invasive pattern, and in high collagen concentration (top right figure), a more invasive pattern. Our computational simulation results (bottom right figure with 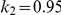 and bottom left figure with 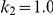) are comparable to the experimental data. The simulation results were taken at 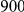 MCS. Reprinted from Biophysical Journal, 89/1, L. Kaufman, C. Brangwynne, K. Kasza, E. Filippidi, V. Gordon, T. Deisboeck, and D. Weitz, Glioma expansion in collagen I matrices: analyzing collagen concentration-dependent growth and motility patterns, 635–650, Copyright (2005), with permission from Elsevier [OR APPLICABLE SOCIETY COPYRIGHT OWNER].

Our simulation results have verified *in vitro* and *in vivo* experiments, that the level of invasiveness of tumour cells can be assessed from the extent of the loss of cell-cell adhesion. We can see in our simulations that high level of invasiveness is achieved by down-regulation of cell-cell adhesion, that is by decreasing the values of 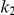. We use 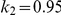 to simulate more invasive scenario and 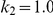 for less invasive scenario as shown by the bottom right and bottom left figures in Fig. 11, respectively. This then must be followed by up-regulation of cell-matrix adhesion, another component that is required for successful invasion. This “discrete analogy” can be related to the inverse relation between cell-cell and cell-matrix adhesion, that is in order to invade and migrate through the surrounding tissue, cell-cell adhesion should be sufficiently low and cell-matrix adhesion should be sufficiently high.

In the study by [31] of glioblastoma multiforme (GBM) growth and invasion, it was shown the effects of increasing collagen concentration on the level of invasiveness of GBM cells, which is similar to increasing cell-matrix adhesion. GBM implanted in a high collagen concentration at early times shows growth patterns typical of malignant tumours where invasive cells gradually accumulate from the centre of MTS, invading outwardly in all directions, as shown in the top right figure of Fig. 11 of the experimental data. On the other hand, GBM that has been implanted in a low collagen concentration shows relatively few invasive cells that tend to invade along distinct branches, as shown in the top left figure. We can relate this to invasion assay simulations that we have performed using a slow 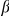-catenin degradation rate 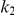 (where a low 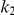 value implies more invasive tumour cells). Here, we compare the results of our simulations in Fig. 11 with experimental data from [31]. Using 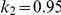 to simulate the invasive scenario and 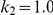 to simulate the less invasive scenario, our simulations show different growth patterns of MTS that are strikingly noticeable between MTS with more invasive cells (bottom right figure) and MTS that is less invasive (bottom left figure). Qualitatively, our simulations are comparable to the experimental data.

Although we cannot directly compare our simulation results (bottom right and left figures) with the experimental results of [31] (top right and left figures), the patterns of invasion from decreasing cell-cell adhesion (our simulation results) show similarities with the patterns of invasion from increasing cell-matrix adhesion (experimental results). We note that GBM is a sarcoma and likely not use E-cadherin/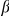-catenin signalling as it is not originated from epithelial tissues. Instead, sarcomas along with other types of brain tumours, express N-cadherin that also mediate calcium-dependent intercellular adhesion. Nevertheless, another paper by [18] developed another multiscale model of transendothelial migration (TEM) involving N-cadherin in which the pathway that they developed is not far different than the pathway using E-cadherin, based on their literature study. Hence, there may be possibility that the kinetics of intracellular proteins of GBM similar to the kinetics we have described here.

## Discussion

In this paper, we have developed a multiscale individual cell-based model to study the roles of intracellular E-cadherin and 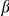-catenin dynamics in cell-cell adhesion within tumours and tumour cell invasion. To model the intracellular biology, we used a mathematical model developed by [14]. We used CC3D, a lattice-based simulation environment, for modelling the cellular level and Bionetsolver, a programming library, for modelling the subcellular (or intracellular) level. The integration of CC3D and Bionetsolver modelling tools enables us to study cell behaviours that are driven by the dynamics inside cells. It allows us to tune the level of detail at the intracellular level, without switching the simulation framework, and examine the effects of changing details at the cellular level.

In the model presented here, we examined invasive behaviours of cancer cells by modifying key parameters that are responsible for cell adhesion. Studies have suggested that nuclear 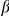-catenin upregulation may characterise invasive cell populations in many types of cancer [29], [32]–[34]. It is possible to tune parameters that regulate the concentration of free 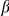-catenin (including nuclear 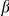-catenin) to study cancer invasiveness *in silico*. Two parameters considered by [14] and that we considered in our simulations are 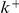 and 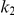. The parameter 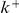 influences the association rate of 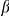-catenin with the proteasome. A sufficiently high 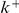 helps maintain appropriate cell-cell adhesion and tumour compactness because it keeps the 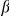-catenin concentration of all cells well below a threshold value. However, when 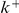 is decreased to a sufficiently low value, free 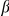-catenin accumulates in the cytoplasm as a result of decreased 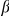-catenin-proteasome complex. This leads to EMT events, in which cells lose cell-cell adhesion, break off from the primary tumour body, and migrate through and invade surrounding tissue. Varying the parameter 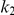 (which influences the rate of 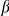-catenin degradation) affects the invasive potential of cells as demonstrated by our simulated invasion assays. Sufficiently low 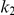 results in cells that are more invasive than cells with a comparatively high 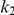. Our simulation results obtained by varying 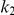 are qualitatively comparable to experimental data obtained in a study of multicellular tumour spheroids.

**Figure 12:**
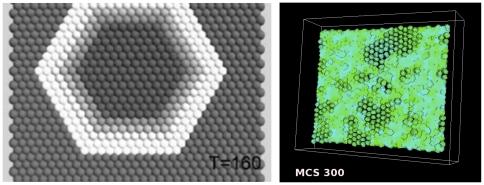
Comparison of 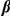-catenin detachment wave simulations based on the centre model of [14] (left figure) and our CC3D-Bionetsolver simulation results (right figure).

While we were able to qualitatively reproduce results from [14], there were noticeable discrepancies that are likely due to fundamental differences in the two simulation methodologies. In contrast to the centre-based implementation of [14], where it is possible to manipulate a single cell and thereby initiate detachment waves, our CC3D-BionetSolver framework does not easily permit a similar level of control. In other words, it was difficult to control cell properties in such a way to cause detachment waves to appear from a single cell in an epithelial layer and propagate radially outward in a regular manner (as shown in Figures 5 and 6 in the paper by [14]). Instead, by reducing 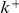 from 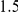 to 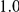 in our GGH model, detachment waves randomly arose from localised groups of cells within epithelial cell layers and propagated outward irregularly. The discrepancies indicate that our approach and the centre-based approach are quantitatively different which was one of the aims of this paper. Nevertheless the results are qualitatively the same. See Fig. 12 for a comparison.

Another difference, is that the stochastic nature of the GGH model results in fluctuations of intracellular variables (concentrations) because of the fluctuating contact areas between cells. This can be seen from the plots of concentration data shown in Figs. 3 and 4. It should be noted that the question of what fundamental differences exist between these two simulation methodologies is distinct from the question of how well the simulation results collectively (of either methodology) reflect or correspond to actual experimental observations. This latter issue, while centrally important in the field of biological modelling, does not fall within the scope of the current study. Primary contributions of our study include the following: (1) It brings to light important differences that exist between two major individual cell-based modelling methodologies (the centre-based model and the GGH model) within the context of cancer biology and (2) it provides an introduction to CC3D-Bionetsolver, a recently developed multiscale framework for multicellular simulation.

## Methods

### 0.7 Glazier-Graner-Hogeweg or GGH Model

The GGH model contains description of objects (*e.g.*, cells, ECM, diffusible fields), interactions (*e.g.*, cell-cell adhesion, morphogen-dependent cell growth), initial conditions (*e.g.*, initial configuration of cells based on a time-lapse microscopy image), and the time evolution of cell properties (*e.g.*, 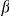-catenin concentration dynamics driving adhesive cell properties or rule-based cell type differentiation).

**Figure 13:**
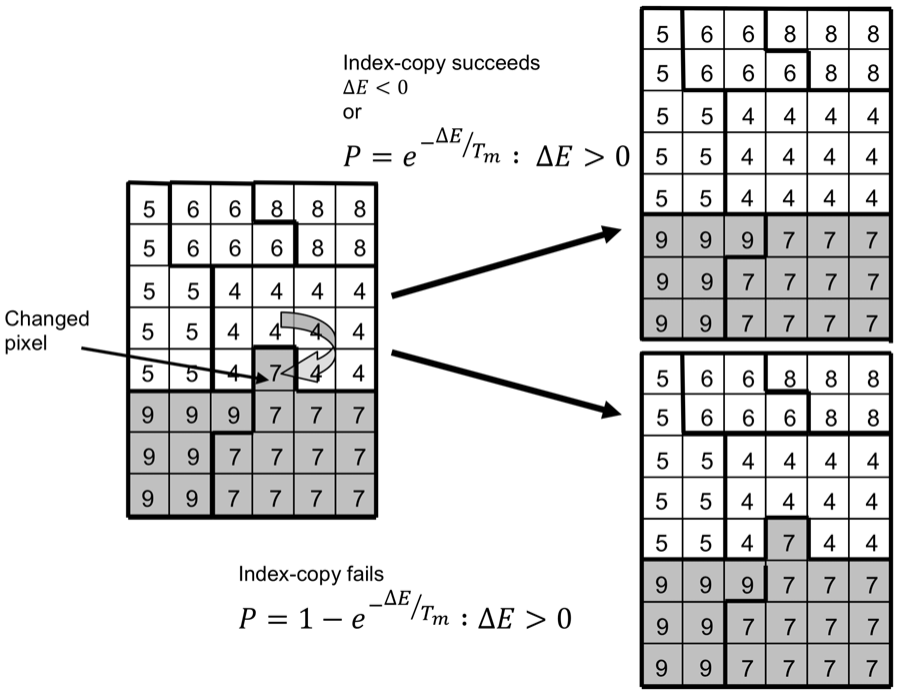
Schematic diagram showing the GGH representation of an index-copy attempt for two cells on a 2-dimensional square lattice. The “white” pixel (source) of cell with 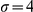 attempts to replace the “grey” pixel (target) of cell with 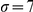. The probability of accepting the index copy is given by equation (1). Bold lines denote boundaries of the cells. Pixel colour denotes cell type. Notice that in GGH simulations we typically have multiple cells with different id 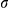 but belonging to the same type 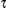.

In the GGH model cells are represented as spatially extended domains on a fixed lattice, usually 3D Cartesian lattice or 3D hexagonal lattice. Each cell is simply a collection of lattice pixels having the same index (also referred to as cell id) 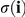 where 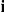 denotes lattice pixel, see Fig. 13. The GGH also allows compartmentalised cells where domains represented cells are further subdivided into subcompartments representing distinct parts of a biological cells (*e.g.*, membrane, organelles, etc) [35].

The dynamics of cells in the GGH model is described by effective energy formalism and implemented as a Monte Carlo algorithm. At each step we randomly select a pixel 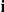 as a target pixel and randomly select one of its neighbouring pixels 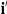 (in this paper we use consider pixels up to fourth nearest neighbour) as a source pixel. Then we attempt to change its index from 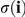 to the index of 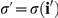. For each pixel copy attempt, we calculate the change in the overall system effective energy 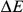 and accept the attempted pixel reassignment with probability 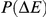:
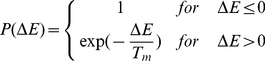
(1)where 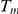 is a parameter representing the effective cell motility. If 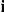 and 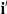 belong to the same cell i.e. when 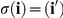 we do not copy the index.

The net result of this algorithm is that the cellular pattern in the GGH model evolves to minimise effective energy. We use this property of the GGH model to construct energy terms in such a way that their minimisation mimics actual cellular behaviour.

The simulation is subdivided (temporally) into so-called Monte Carlo Steps (MCS) which correspond to a unit of physical time. By convention, each MCS consists of number of pixel copy attempts equal to the total number of lattice sites. The conversion between pixel and physical distance (or MCS and physical time) depends on model parameters. In a simple case for example, in Bionetsolver we set timestepBionetwork to 0.03 and if Bionetsolver gets called every MCS then 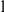 MCS corresponds to 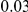 hours. In this paper we do not specifically set a relationship between MCS and the physical time because in the computational simulations we also incorporate cell mitosis or cell division which in the process itself also requires another time convention. In the mitosis process we do not apply any intracellular pathway, but instead we use a built-in mitosis function provided by CC3D.

The physical distance is recovered by converting pixels into units of length. This conversion is more straightforward than the correspondence between MCS and physical time and in our simulations we set 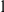 pixel to correspond to 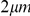.

The effective energy, also called the Hamiltonian and denoted by either 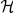 or 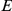, is the core of the GGH model. The Hamiltonian is typically expressed as a sum of terms, each term representing different cellular behaviours, interactions, mechanics, etc. The effective energy mixes true energies such as cell-cell adhesion with terms that mimic energies (*e.g.*, the response of a cell to a chemotactic gradient of a chemical field). In our simulations we have used Hamiltonian containing adhesion energy term, two terms implementing constraints on cellular shapes (volume and surface) and one term implementing chemotactic force:
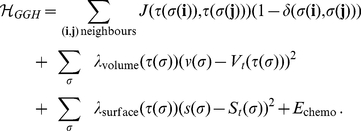
(2)


The first term of Hamiltonian in Eq. 2 represents variations in energy due to adhesion between cells of different types, a *boundary energy*, that depends on 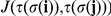 between two cells 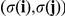 of given cell types 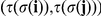 at a link (the interface between two neighboring pixels). The sum is over all neighbouring pairs of lattice sites 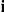 and 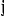 (note that the neighbour range may be greater than one), the boundary energy coefficients are symmetric,
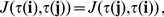
(3)and
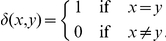
For cell volume, most GGH simulations employ a volume constraint that restricts volume variations of generalised cells from their target volumes, defined as
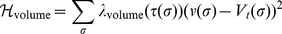
(4)where for an individual cell 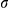, 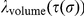 denotes the inverse compressibility of the cell, 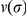 is the number of pixels in the cell (cell volume), and 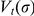 is the cell's target volume. One useful result from the constraint formalism is that it defines 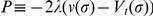 as the pressure inside the cell. If 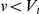 the cell has a positive internal pressure while if 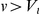 the cell has a negative pressure.

In an analogous way we implement constraint on cell surface which is motivated by the fact that cells have (almost) fixed amounts of cell membrane. A surface area constraint can be defined
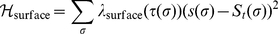
(5)where 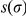 is the surface area of cell 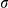, 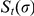 is cell's target surface area, and 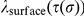 is cell's inverse membrane compressibility.

The chemotaxis term 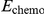 represents interaction of cells with external chemical gradient 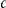. It is easier, and somewhat less confusing to actually write expression for 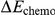 than for 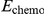. For a given pixel copy attempt we define change in the chemotaxis energy term as:
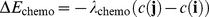
(6)where 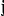 denotes target pixel and 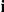 denotes source pixel for an attempt to copy 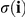 to pixel 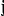.

Strictly speaking 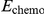 is quasi energy term which is used to produce biased cell motion up (or down) the concentration gradient depending on sign of 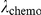. The 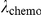 determines how strong a given cell responds chemotactically to the external gradient 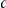.

In Supporting Information S1 we provide a brief CC3D tutorial which covers the basic usage of the GGH model. More detailed information about the model can be found in [28], [36], [37] and tutorial documentation on http://www.compucel3d.org/Manual http://www.compucel3d.org/Manual.

### 0.8 Bionetsolver programming library

Bionetsolver is a C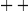 library with a high-level Python API that permits easy definition of sophisticated models coupling reaction-kinetic models described in the SBML with GGH objects for execution in CompuCell3D. Bionetsolver makes use of the SBML ODE Solver Library (SOSlib) to implement reaction-kinetic network dynamics which can regulate the cell dynamics generated by the GGH core. For further information on SOSlib, the reader may refer to the paper in [38]. SOSlib provides functionality both for reading SBML models and solving them as a system of ODEs. In addition to this functionality, there are three classes – BionetworkSBML, BionetworkTemplateLibrary and Bionetwork – that provide some additional convenience in storing and manipulating SBML models as well as creating ODE integrators and time-stepping the integrators.

The Python API of Bionetsolver provides a set of 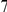 core functions that can be called from within a CC3D Steppable. These 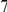 functions are used for initialisation and manipulation of Bionetsolver objects from within the steppable. In this way, the entire specification of a multiscale (cell-subcell-level) simulation can be written in Python and executed in the CC3D player.

After the Bionetsolver API is imported and initialised, SBML models are loaded with a loadSBMLModel function and each SBML model can be added to one or more template libraries using the function addSBMLModelToTemplateLibrary. When loadSBMLModel is called, a string argument is required that signifies a name for the SBML model. Similarly, when addSBMLModelToTemplateLibrary is called, the user provides the SBML model name (specified when loadSBMLModel was called) as well as a string argument that signifies the name of the template library. A single SBML model may be added to several template libraries and each template library may contain one or more SBML models. The code example below shows the use of these functions. Both of them are called from within the start function of a CC3D steppable. Notice that when loading an SBML model, both a model name and a model key are provided as arguments. The model key is used to reference specific SBML models in certain function arguments. In the example below, a single SBML model is loaded and is added to two bionetwork templates, “LowBetaCat” and “HighBetaCat”. To associate bionetworks with CC3D cells, the template name must be the name of a CC3D cell type.

*# Create a bionetwork SBML model named SimpleModel*

sbmlModelName="SimpleModel”

sbmlModelKey="SM”

sbmlModelPath = os.getcwd()+“/MultiScaleModels/sbmlModels/SimpleExample.sbml”

bionetAPI.loadSBMLModel(sbmlModelName, sbmlModelPath, sbmlModelKey)

*# Add “SimpleModel” to templates called “LowBetaCat” and “HighBetaCat”*

bionetAPI.addSBMLModelToTemplateLibrary(“SimpleModel”, “LowBetaCat”)

bionetAPI.addSBMLModelToTemplateLibrary(“SimpleModel”, “HighBetaCat”)

In addition, a setBionetworkInitialCondition function can be used to specify initial conditions for parameters and state variables in any SBML model within a template library. As arguments to this function, the user provides a template library name, a variable or parameter name and the corresponding initial numerical value of the variable or property. As indicated above, a set of SBML models stored within a template library can be associated with a CC3D cell type by providing the cell type name as the name of the template library. When the function initializeBionetworks is called, a separate bionetwork object is created for each cell of the given cell type and the previously specified initial conditions (specified using setBionetworkInitialCondition) are set for each of the bionetworks. Any parameters or state variables for which setBionetworkInitialCondition was not called are simply initialised, by default, to values specified in the original SBML models. A code excerpt below shows the use of these functions. Note that the variable name provided as the second argument to setBionetworkInitialCondition is prefixed with the SBML model key (in this case SM for SimpleModel). In case the same variable or parameter name, k1, appears in another SBML model in the same template library, this prefix uniquely identifies the parameter k1 found in the model indicated by the key SM. The initializeBionetworks function accepts a single numerical argument which corresponds to the timestep length used in the simulation to update the SBML models.

*# Set initial conditions for templates “LowBetaCat” and “HighBetaCat”*

bionetAPI.setBionetworkInitialCondition(“LowBetaCat”, “SM
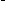
k1”, 0.9

bionetAPI.setBionetworkInitialCondition(“HighBetaCat”, “SM
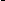
k1”,0.1

*# Create bionetwork instances from templates and initialise states*

initializeBionetworks(0.05)

All of the functions mentioned above are initialisation functions and are called within the start function of the CC3D steppable. In addition, there are three more functions that are called within the step function of the CC3D steppable. These are (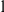) timestepBionetworks, for time-stepping the ODE integrators, (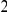) getBionetworkValue, for retrieving SBML property and state variable values from the integrators, and (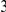) setBionetworkValue, for setting SBML property values of the integrators. The code excerpt below shows an example of how getBionetworkValue and setBionetworkValue can be called inside a ÔforÕ loop that iterates over CC3D cells. The cell ID (referenced by cell.id) is used to retrieve and set variable and parameter values for bionetworks (*i.e.*, SBML models) associated with each cell.

for cell in self.cellList:

S1 = bionetAPI.getBionetworkValue(“SM
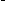
S1”, cell.id)

bionetAPI.setBionetworkValue(“SM
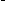
S1”, 0.1, cell.id)

CC3D cell-level properties can be retrieved using procedures described in the CC3D documentation and the CC3D demo simulations. Finally, SBML property values can be set as a function of CC3D cell properties and, likewise, CC3D cell properties can be set as a function of SBML state variable values. This is how mechanistic coupling can be established between SBML (subcellular) and CC3D (cell-level) properties and dynamics.

Finally, Bionetsolver has a function copyBionetworkFromParent that can be used inside the updateAttributes function of a CC3D mitosis steppable to copy a parent cell bionetwork to a child cell that has just undergone mitosis. Copies of the parent SBML model integrators are created and the states of the original integrators are copied to the new integrators. The use of this function is illustrated in the code excerpt below.

class MitosisSteppable(MitosisSteppableBase):

def 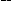
init
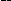
(self, 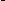
simulator, 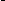
frequency = 1):

MitosisSteppableBase.
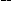
init
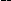
(self, 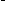
simulator, 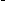
frequency)

updateAttributes(self):

childCell = self.mitosisSteppable.childCell

parentCell = self.mitosisSteppable.parentCell

bionetAPI.copyBionetworkFromParent(parentCell, childCell)

### 0.9 E-cadherin and 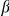-catenin kinetics

**Figure 14:**
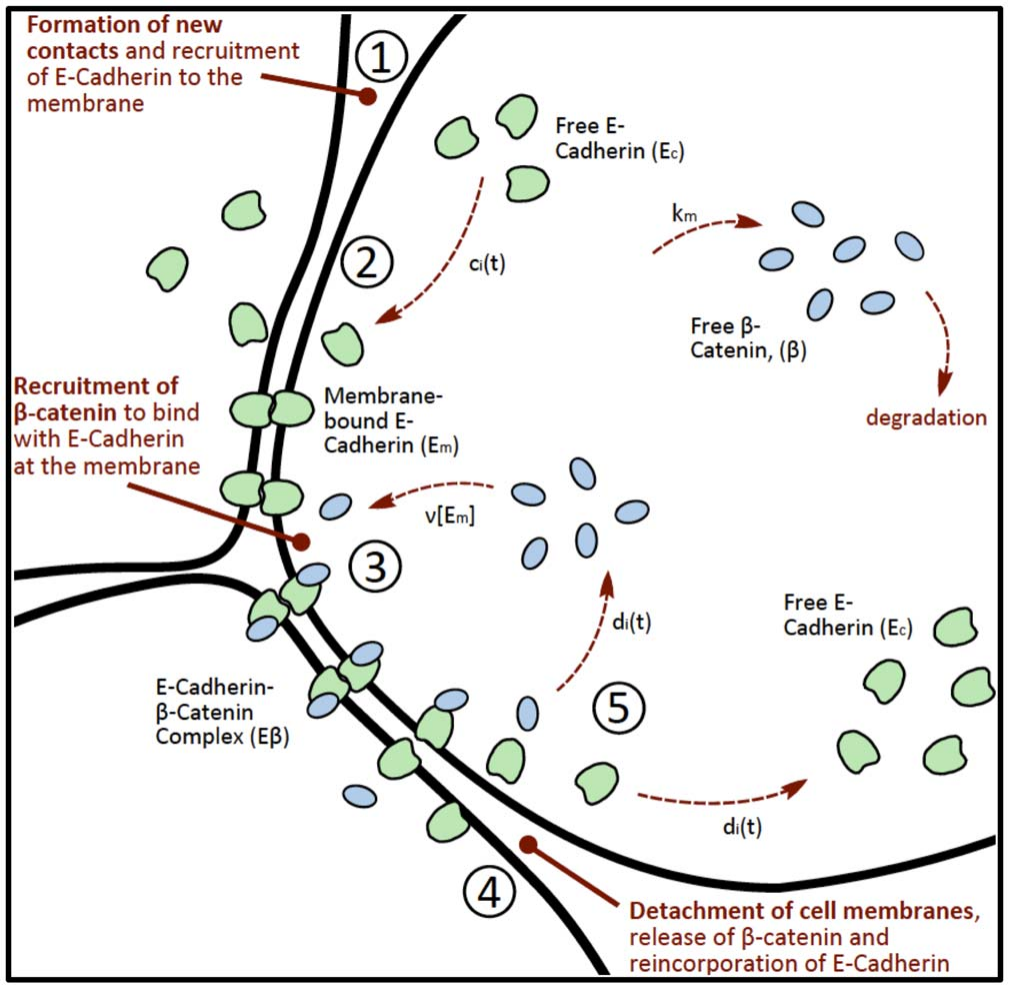
A schematic diagram of the E-cadherin and interactions with 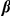-catenin.

In [14], the kinetics of E-cadherin and 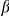-catenin in a cell are conceptually modelled as follows. After being synthesised, E-cadherin is released to the cytoplasm as free E-cadherin (the concentration is denoted by 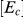). Free 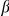-catenin (concentration 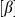) is assumed to be distributed in the cytoplasm and near the cell membrane. When there is signalling for cell-cell contact, free E-cadherin in the cytoplasm (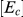) is transported to the cell membrane (concentration 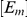) where its cytoplasmic domain binds to free 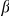-catenin to form E-cadherin-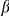-catenin complex (concentration 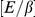) and the extracellular domain binds to E-cadherin-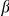-catenin complex of adjacent cells. If cell detachment occurs, E-cadherin-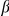-catenin complex dissociates, releasing free 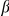-catenin and sequestering E-cadherin into the cytoplasm by endocytosis. The free 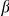-catenin is then degraded and downregulated after binding with proteasome. These intracellular interactions are summarised in a schematic diagram shown in Fig. 14.

The mathematical model we use to describe the dynamics of these key chemical species concentrations (including the component influencing cell-cell adhesion *i.e.*, E-cadherin-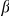-catenin complex) in each individual cell 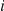 formulated precisely as in [14] *i.e.*

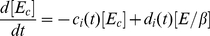
(7a)

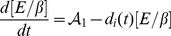
(7b)

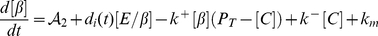
(7c)

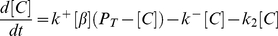
(7d)where, depending on signalling,
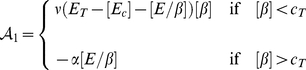
(8)and
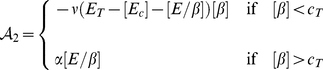
(9)


The parameter 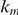 is the rate of production of 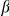-catenin, 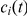 is a time-dependent function describing the amount of cadherin stimulated to form bonds in response to increased cell-cell contact, and 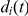 is a function that describes the amount of cadherin released as a result of broken bonds during cell detachment. These functions (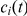 and 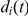) depend on the rate of change in contact area between adjacent cells. They are defined as follows:
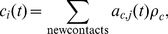
(10)and
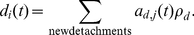
(11)where 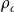 and 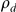 are rate constants associated with E-cadherin translocation between the cell membrane and the cytosol. The rate constant 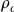 reflects how quickly E-cadherin is transported from the cytoplasm to the cell membrane in response to cell-cell contact signalling and 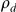 reflects the rate of the reverse action (from the membrane to the cytoplasm) when cell detachment occurs. In [14], 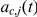 and 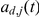 are time-dependent functions quantifying the rate of increase in contact area (when E-cadherin is transported from the cytosol to the membrane) and the rate of loss of contact area (when E-cadherin is reincorporated from the membrane to the cytosol), respectively. These functions are defined as follows [14]:
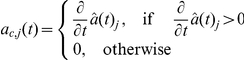
and
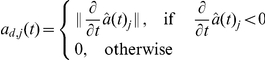
where 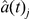 is the approximated contact area between cells 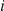 and 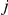 at time 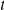 divided by the surface area of cell 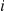.

The condition for attachment is assumed valid as long as the concentration of free 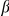-catenin 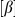 is below a threshold value, 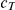. For detachment to occur, the amount of free 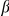-catenin in the cytoplasm must be sufficiently high with an additional contribution from dissociated E-cadherin-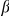-catenin complex. In other words, free 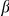-catenin must be higher than the threshold value (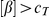). This claim is based on several studies that have found upregulation of 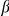-catenin in the cytoplasm (or termed nuclear 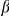-catenin) at the invasive front of colorectal carcinomas [29], [33], invasive breast cancers [34], fibrosarcoma, clear cell sarcoma and carcinosarcoma [32]. Nuclear accumulation of 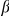-catenin initiates the loss of epithelial differentiation and gain of mesenchyme-like capabilities of the tumour cells at the invasive front, while in the central areas of the primary tumours, nuclear 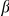-catenin was found to be localised to the cell membrane. Nuclear accumulation of 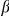-catenin has been the most powerful predictor of liver metastasis in colorectal cancer. This may be an important marker for adjuvant therapy or other treatment modalities.

In order to obtain a nondimensional system of equations, we nondimensionalise Eqs. (7a)–(7d) in the usual way by setting the following dimensionless variables:
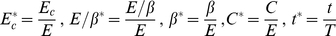
where 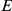 is a reference concentration of E-cadherin and 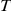 is an appropriate reference time, from which we obtain dimensionless parameters:
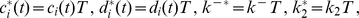


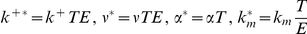



Inserting the dimensionless variables and parameters into the system (7) and after dropping the asterixes for notational convenience, we obtain the dimensionless system of equations
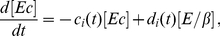
(12a)

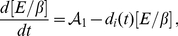
(12b)

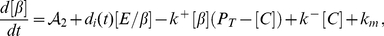
(12c)

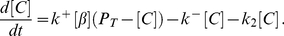
(12d)


The dimensionless parameter values used in the simulations of this chapter can be found in Table 1. These values are based on the parameters used in [14] where we nondimensionalise the parameters by assuming 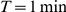 and 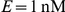, unless stated otherwise.

## Supporting Information

Supporting Information S1 Information about how to configure, run, and modify two simple CC3D GGH-based simulations.
(DOCX)
