# Supplementary material for: Integrating Intracellular Dynamics Using CompuCell3D and Bionetsolver: Applications to Multiscale Modelling of Cancer Cell Growth and Invasion
Source: PLoS One. 2012 Mar 26;7(3):e33726. doi: 10.1371/journal.pone.0033726 (PMC3312894; doi:10.1371/journal.pone.0033726)
Supplement: Supporting Information S1 — Information about how to configure, run, and modify two simple CC3D GGH-based simulations. (DOCX) [file pone.0033726.s001.docx]

**S1: Supporting Information**

**for**

**“Integrating Intracellular Dynamics using CompuCell3D and Bionetsolver: Applications to Multiscale Modelling of Cancer Cell Growth and Invasion”**

Vivi Andasari^1*^, Ryan T. Roper^2^, Maciej H. Swat^3^, Mark A. J. Chaplain^1^

1 Division of Mathematics, University of Dundee, Dundee DD1 4HN, Scotland, UK

2 was at the Computational Systems Biology/Sauro Lab, University of Washington,

Seattle, Washington, USA

3 Biocomplexity Institute and Department of Physics, Indiana University, Bloomington

IN, 47405-7105, USA

*To whom correspondence should be addressed.

Email: [**vivi@maths.dundee.ac.uk**](mailto:vivi@maths.dundee.ac.uk)

# Supporting Information

GGH models in CompuCell3D (or CC3D) simulation framework are described using a combination of the CompuCell3D Markup Language (CC3DML) and Python scripting. Such a combined approach allows one to build complex biomedical models and does not require recompilation when running them. In a typical CC3D simulation, “static” aspects of the model such as lattice size, simulation runtime, list of cell types, initial conditions or cadherin affinities, are usually described using CC3DML. We can replace CC3DML with equivalent Python syntax. The “dynamic” part of the CC3D model is described using Python scripting. Since Python is a full-featured programming language, modelers are able to express complex cell type differentiation rules, couple cell properties to concentrations of diffusive chemicals or to cell-cell signaling or parameterize cell adhesive properties in terms of underlying molecular or gene regulatory networks.

In this brief tutorial we will show how to configure, run, and modify two simple CC3D GGH-based simulations: cell sorting and bacterium macrophage system.

## Cell Sorting

Cell sorting due to differential adhesion between cells of different types is one of the basic mechanisms creating tissue domains during development and wound healing and in maintaining domains in homeostasis. In a classic *in vitro* cell sorting experiment to determine relative cell adhesivities in embryonic tissues, mesenchymal cells of different types are dissociated, then randomly mixed and reaggregated. Their motility and differential adhesivities then lead them to rearrange to reestablish coherent homogenous domains with the most cohesive cell type surrounded by the less cohesive. The simulation of the sorting of two cell types was the original motivation for the development of GGH methods.

The easiest way to configure CC3D simulations is to use code-generation capabilities of **Twedit++** - a text editor, which is a part of CC3D suite. We will explain steps necessary to use Twedit++ functionality to generate simulation code and will walk reader through the entire code explaining all concepts in the code. Twedit++ will generate basic simulation code template, which can be further customised. After opening Twedit++ we invoke the simulation wizard to create a simulation, by clicking **CC3DProject -> New CC3D Project** in the menu bar. In the initial screen we specify the name of the model (cellsorting), its storage directory (**C:\CC3DProjects**) and whether we will store the model as pure CC3DML, Python and CC3DML or pure Python. This tutorial will use Python and CC3DML. We can obviously manually type all simulation code, however, using automatic code generators greatly reduces modelers’ effort.


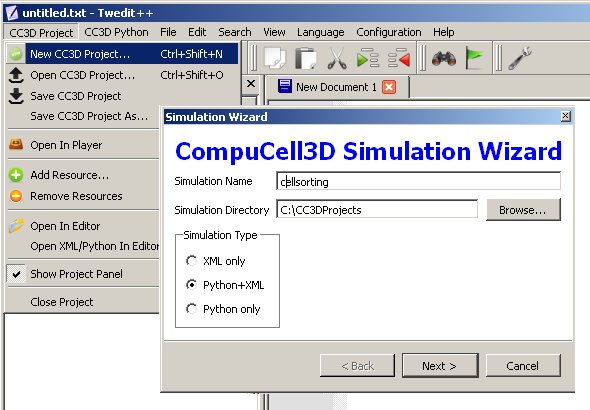


**Supplemental Figure S1.** **Invoking the CompuCell3D Simulation Wizard from Twedit++.**

On the next page of the Wizard we specify GGH global parameters, including cell-lattice dimensions, the cell fluctuation amplitude (also known in the literature as Temperature or *T*), the duration of the simulation in Monte-Carlo steps and the initial cell-lattice configuration.

In this example, we specify a 100x100x1 cell-lattice (a 2D model), fluctuation amplitude of 10, simulation duration of 10000 MCS, and a pixel-copy range of 2. BlobInitializer initializes the simulation with a disk of cells of specified size.


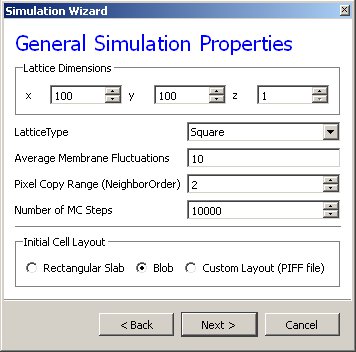


**Supplemental Figure S2.** **Specification of basic cell-sorting properties in Simulation Wizard.**

On the next Wizard page we name the cell types in the model. We will use two cells types: Condensing (more cohesive) and NonCondensing (less cohesive). CC3D by default includes a special generalized-cell type Medium with unconstrained volume which fills otherwise unspecified space in the cell-lattice.


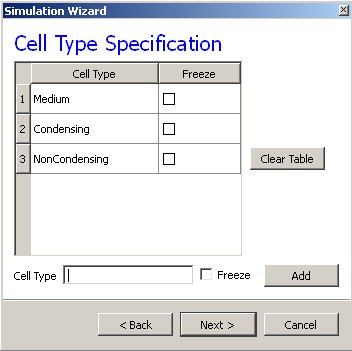


**Supplemental Figure S3.** **Specification of cell-sorting cell types in Simulation Wizard.**

We skip the Chemical Field page of the Wizard and move to the Cell Behaviors and Properties page. Here we select the biological behaviors we will include in our model. Since cell sorting depends on differential adhesion between cells, we select the Contact module from the Adhesion section (see Figure 4) and give the cells a defined volume using constraint Volume from Constraints and Forces section.

**
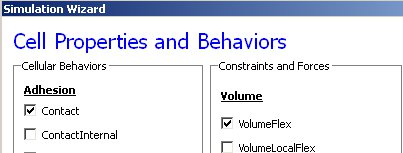
**

**Supplemental Figure S4. Selection of cell-sorting cell behaviors in Simulation Wizard.**^[[1]](#footnote-1)^

We skip the next page related to Python scripting, after which Twedit++-CC3D generates the draft simulation code. Double-clicking on **cellsorting.cc3d** opens both the CC3DML (**cellsorting.xml**) and Python scripts for the model. Because the CC3DML file contains the complete model in this example, we postpone discussion of the Python script. A CC3DML file has 3 distinct sections. First, the *Lattice Section* (lines 2-7) that specifies global parameters, like cell-lattice size. The *Plugin Section* (lines 9-32) lists all the plugins used, *e.g.,* CellType and Contact. The *Steppable Section* (lines 34-41) lists all steppables (modules which run every Monte Carlo Step or with user specified frequency), here we use only BlobInitializer which runs only at the beginning of the simulation and lays out cells on the lattice.

1. <CompuCell3D version="3.6.0">
2. <Potts>
3. <Dimensions x="100" y="100" z="1"/>
4. <Steps>10000</Steps>
5. <Temperature>10.0</Temperature>
6. <NeighborOrder>2</NeighborOrder>
7. </Potts>
8. <Plugin Name="CellType">
9. <CellType TypeId="0" TypeName="Medium"/>
10. <CellType TypeId="1" TypeName="Condensing"/>
11. <CellType TypeId="2" TypeName="NonCondensing"/>
12. </Plugin>
13. <Plugin Name="Volume">
14. <VolumeEnergyParameters CellType="Condensing" LambdaVolume="2.0”
15. TargetVolume="25"/>
16. <VolumeEnergyParameters CellType="NonCondensing" LambdaVolume="2.0"
17. TargetVolume="25"/>
18. </Plugin>
19. <Plugin Name="CenterOfMass"/>
20. <Plugin Name="Contact">
21. <Energy Type1="Medium" Type2="Medium">10</Energy>
22. <Energy Type1="Medium" Type2="Condensing">10</Energy>
23. <Energy Type1="Medium" Type2="NonCondensing">10</Energy>
24. <Energy Type1="Condensing"Type2="Condensing">10</Energy>
25. <Energy Type1="Condensing" Type2="NonCondensing">10</Energy>
26. <Energy Type1="NonCondensing" Type2="NonCondensing">10</Energy>
27. <NeighborOrder>2</NeighborOrder>
28. </Plugin>
29. <Steppable Type="BlobInitializer">
30. <Region>
31. <Center x="50" y="50" z="0"/>
32. <Radius>20</Radius>
33. <Width>5</Width>
34. <Types>Condensing,NonCondensing</Types>
35. </Region>
36. </Steppable>
37. </CompuCell3D>

**Supplemental List L1.** **Simulation-Wizard-generated draft CC3DML (XML) code for cell-sorting.**^[[2]](#footnote-2)^

All parameters appearing in the autogenerated CC3DML script have default values inserted by Simulation Wizard. We must edit the parameters in the draft CC3DML script to build a functional cell-sorting model (Supplemental List **L1**). The CellType plugin (lines 9-13) already provides three generalized-cell types: Condensing (C), NonCondensing (N) and Medium (M), so we need not change it.

However, the boundary-energy (Contact-energy) matrix in the Contact plugin (lines 24-32) is initially filled with identical values, *i.e.*, the cell types are identical. For cell-sorting, Condensing cells must adhere strongly to each other (so we set *J_CC_=2*), Condensing and NonCondensing cells must adhere more weakly (here we set *J_CN_=11*) and all other adhesion must be very weak (we set *J_NN_=J_CM_=J_NM_=16*), as discussed in [1,2,3]. The value of *J_MM_=0* is irrelevant, since the Medium generalized cell does not contact itself. When choosing contact energies it is useful to use the following rule of thumb: two cells will try to stick to each other if the contact energy between them is low and two cells will avid contact if the contact energy between them is high, where “low” and “high” have relative meaning.

To reduce artifacts due to the anisotropy of the square cell-lattice we increase the neighbor-order range in the contact energy to 2 so the contact-energy sum in Equation (2) in the main manuscript will include nearest and second-nearest neighbors (line 31).

In the Volume plugin, which calculates the Volume-constraint energy given in Equation (4) the attributes CellType, LambdaVolume and TargetVolume inside the <VolumeEnergyParameters> tags specify *λ(τ)* and *V_t_(τ)* for each cell type. In our simulations we set *V_t_(τ)*=25 and *λ(τ)=*2.0 for both cell types.

We initialize the cell lattice using the BlobInitializer, which creates one or more disks (solid spheres in 3D) of cells. Each region is enclosed between <Region> tags. The <Center> tag with syntax

<Center x="x_position" y="y_position" z= "z_position"/> specifies the position of the center of the disk. The <Width> tag specifies the size of the initial square (cubical in 3D) generalized cells and the <Gap> tag creates space between neighboring cells. The <Types> tag lists the cell types to fill the disk. Here, we change the Radius in the draft BlobInitializer specification to 40. These few changes produce a working cell-sorting simulation.

To run the simulation we open CompuCellPlayer and select **cellsorting.cc3d** from the **File -> Open Simulation File** dialog.

Supplemental Figure S5 shows snapshots of a simulation of the cell-sorting model. The less cohesive NonCondensing cells engulf the more cohesive Condensing cells, which cluster and form a single central domain.


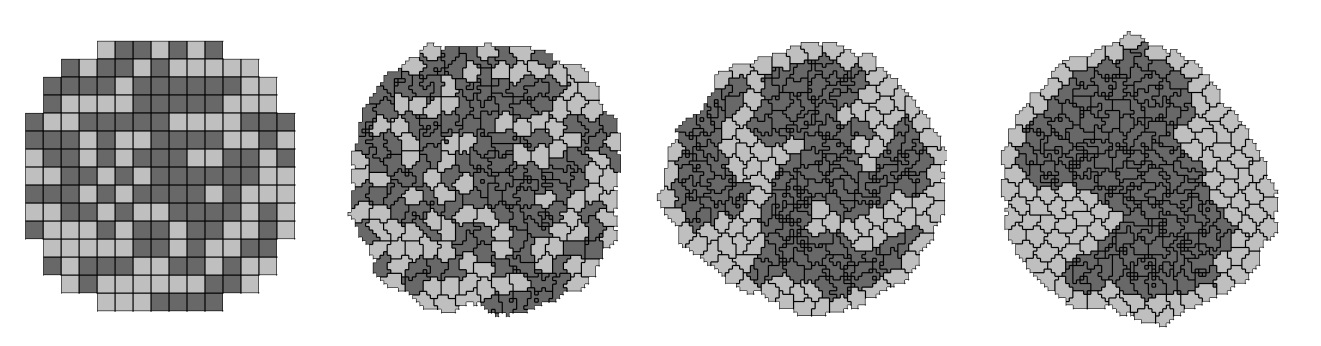


*t*=0 MCS

*t*=20 MCS

*t*=880 MCS

*t*=10000 MCS

**Supplemental Figure S5.** **Snapshots of the cell-lattice configurations for the cell-sorting simulation in Supplemental List L1.** The boundary-energy hierarchy drives NonCondensing (light grey) cells to surround Condensing (dark grey) cells. The white background denotes surrounding Medium.

By changing the boundary energies we can produce other cell-sorting patterns. The easiest modification of the above sorting simulation is to set contact energy between Medium and cells to be low compared to other energies in the energy hierarchy. Setting *J_CC_=J_CN_=J_NN_=11* and *J_NM=_J_CM_=5* (lines 24-32) results in the dissociative cell pattern.


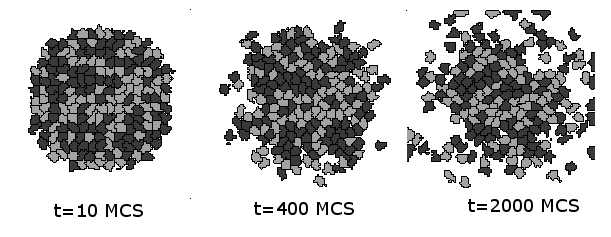
**Supplemental Figure S6.** **Snapshots of the cell-lattice configurations for simulations where contact energy hierarchy leads to dissociative pattern.**

In an analogous way we can produce checkerboard pattern by setting homotypic energies to be high compared to heterotypic energies. When we use the following values *J_CC_=J_NN_=11 J_CN_=*2 and *J_NM_*=*J_CM_*=16 (lines 24-32) we obtain the following pattern:


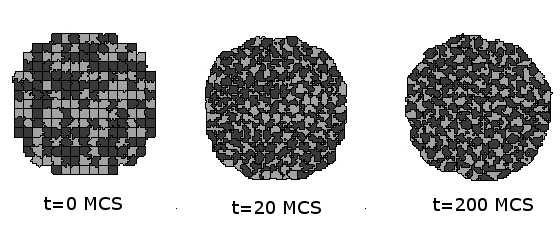
**Supplemental Figure S7. Snapshots of the cell-lattice configurations for simulations where contact energy hierarchy leads to checkerboard pattern.**

To investigate influence of the cell membrane fluctuation amplitude on cellular pattern we run two versions of simulation shown in Supplemental Figure S8 – one with fluctuation amplitude set to 0.1 and one with *T*=100 (line 5). The results are shown below.


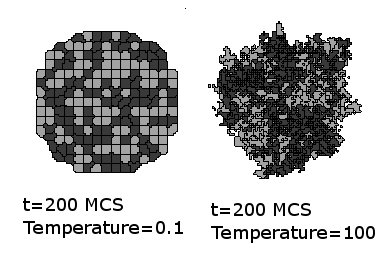


**Supplemental Figure S8. Snapshots of the cell-lattice configurations for simulations run with extreme membrane fluctuation amplitudes (Temperature).**

As one can see using low values of membrane fluctuations produces immotile cells whereas high values of *T* give rise to extremely motile cells with jagged cell boundaries. Both cases show here are extreme and outside “normal” T ranges used in a well-tuned GGH simulation.

So far we have modified only CC3DML simulation file. However, by writing few lines of Python code (by modifying autogenerated Python scripts) we implement very complex cell behaviors and build simulation where parameters are changed on-the-fly without the need of any code recompilation. We will only show here simple example and refer reader to full CC3D tutorial presented in the references.

First we will implement simple cell growth. The way we typically “grow” cells in GGH is by increasing target volume in the volume constraint. To enable cell growth we have to remove lines 16-17 and 18-19 in the Supplemental List L1. We also increase lattice dimension to be 150x150 to make room for growing cells and move center of the cellular blob to the center of the lattice (75,75) - line 36 of Supplemental List L1. By not specifying volume constraints parameters in the CC3DML file we tell CC3D to use volume constraint parameters specific for each cell. Such parameters have to be set using Python script and we do this by modifying autogenerated Python steppable (autogenerated file *cellsortingSteppables.py*):

1. from PySteppables import *
2. import CompuCell
3. import sys
4. class cellsortingSteppable(SteppableBasePy):
5. def __init__(self,_simulator,_frequency=10):
6. SteppableBasePy.__init__(self,_simulator,_frequency)
7. def start(self):
8. for cell in self.cellList:
9. cell.targetVolume=25
10. cell.lambdaVolume=2.0
11. def step(self,mcs):
12. for cell in self.cellList:
13. if cell.type==self.CONDENSING:
14. cell.targetVolume+=1

**Supplemental List L2.** **Modifications of autogenerated Python steppable.** Cell growth is implemented by gradually increasing target volume lines 14 and 15.

In lines 8-11 we set initial values of lambda volume and target volume for each cell in the simulation. In Lines 12-15 we implement cell growth of all Condensing cells. Notice that to check the type of cell type we simply use cell type name from the CC3DML (Condensing) capitalize all letters and use modifier self. to extract variable which holds numerical value of the cell type (line 14). To complete the picture we also show main Python file where the object of class Cellsorting Stepable is created.

1. import sys
2. from os import environ
3. from os import getcwd
4. import string
5. sys.path.append(environ["PYTHON_MODULE_PATH"])
6. import CompuCellSetup
7. sim,simthread = CompuCellSetup.getCoreSimulationObjects()
8. # add extra attributes here
9. CompuCellSetup.initializeSimulationObjects(sim,simthread)
10. # Definitions of additional Python-managed fields go here
11. #Add Python steppables here
12. steppableRegistry=CompuCellSetup.getSteppableRegistry()
13. from cellsortingSteppables import cellsortingSteppable
14. steppableInstance=cellsortingSteppable(sim,_frequency=10)
15. steppableRegistry.registerSteppable(steppableInstance)
16. CompuCellSetup.mainLoop(sim,simthread,steppableRegistry)

**Supplemental List L3.** **Modifications of autogenerated main Python script.** Notice that we have changed steppable call frequency in line 18 to be 10.

Running the simulation produces the following pattern.


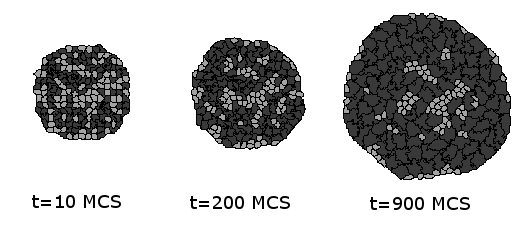
**Supplemental Figure S9.** **Snapshots of the cell-lattice configurations for simulation where target volume of Condensing cells is gradually increasing.** Notice that average size of Condensing cell increases whereas average size of NonCondensing cell does not.

Using Twedit++ we can easily add mitosis module, which would divide growing cells once they reach doubling volume. By right-clicking on **cellsortingSteppables.py** in left project panel of Twedit we select add stoppable called Mitosis (we check appropriate box in the pop up dialog) and Twedit++ pastes code template which we modify to divide only cells of type Condensing once they reach volume 50 (lines 15-19). Immediately after cell division is completed CC3D will run updateAttributes function where we halve target volume of the parent cell (line 24) and set type if the offspring cell to be NonCondensing and target volume and lambda volume of the child cell to be the same as the parent cell (lines 26-28).


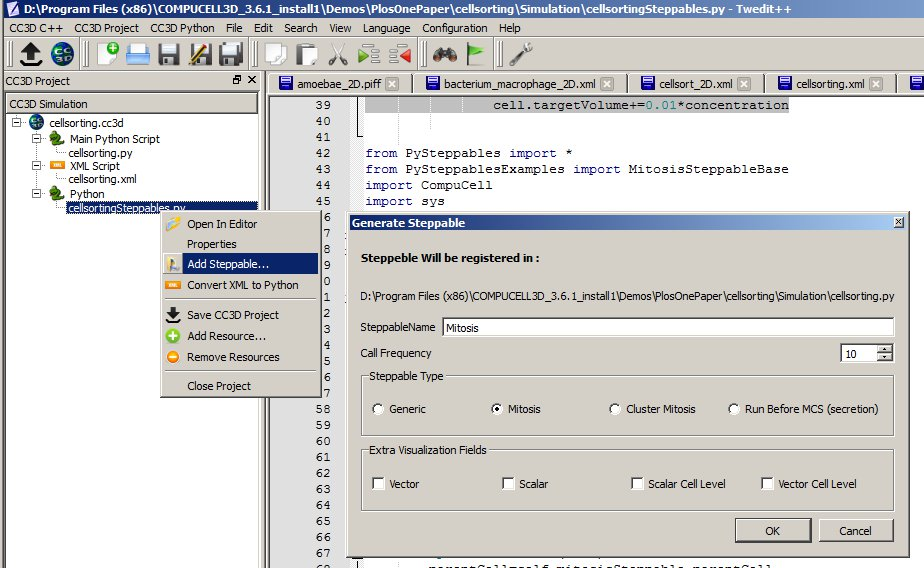


**Supplemental Figure S10.** **Adding mitosis steppable to existing CC3D simulation is greatly facilitated by functionality of Twedit++.**

1. from PySteppables import *
2. from PySteppablesExamples import MitosisSteppableBase
3. import CompuCell
4. import sys
5. from PlayerPython import *
6. from math import *
7. class Mitosis(MitosisSteppableBase):
8. def __init__(self,_simulator,_frequency=1):
9. MitosisSteppableBase.__init__(self,_simulator, _frequency)
11. def step(self,mcs):
12. cells_to_divide=[]
13. for cell in self.cellList:
14. if cell.volume>50 and cell.type==self.CONDENSING:
15. cells_to_divide.append(cell)
16. for cell in cells_to_divide:
17. self.divideCellRandomOrientation(cell)
18. def updateAttributes(self):
19. parentCell=self.mitosisSteppable.parentCell
20. childCell=self.mitosisSteppable.childCell
21. parentCell.targetVolume/=2
22. childCell.type=self.NONCONDENSING
23. childCell.targetVolume=parentCell.targetVolume
24. childCell.lambdaVolume=parentCell.lambdaVolume

**Supplemental List L4.** **Autogenerated MitosisSteppable with custom modification.**

Because after mitosis the offspring cells are of type NonCondensing then as the simulation progresses the ration of NonCondensing cells to Condensing cells will keep increasing as shown in the figure below:


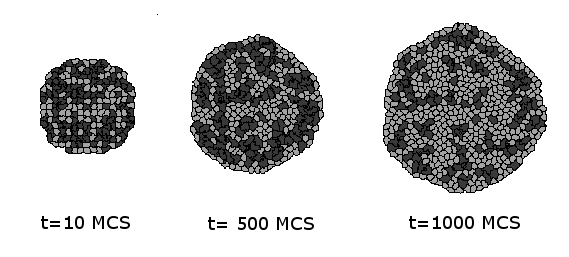


**Supplemental Figure S11.** **Snapshots of the cell-lattice configurations for simulation where target volume of Condensing cells increases but once cell undergo mitosis after reaching doubling volume the target volume is halved.** Notice that average size of Condensing cells is roughly the same as average size of NonCondensing cells.

Finally when we want to couple cellular behaviors to external gradients – *e.g.,* make growth of cells of type Condensing depend on concentration of chemical secreted by NonCondensing cells we introduce diffusible chemical (we modify CC3DML) and from the steppable access the value of concentration field and use it to parameterize cell growth in terms of concentration.

To modify CC3DML we simply insert the following code snippet into Supplemental List L1 right after line 31.

1. <Steppable Type="FlexibleDiffusionSolverFE">
2. <DiffusionField>
3. <DiffusionData>
4. <FieldName>FGF</FieldName>
5. <DiffusionConstant>0.10</DiffusionConstant>
6. <DecayConstant>0.001</DecayConstant>
7. </DiffusionData>
8. <SecretionData>
9. <Secretion Type="NonCondensing">2</Secretion>
10. </SecretionData>
11. </DiffusionField>
12. </Steppable>

**Supplemental List L5.** **Specification of a diffusible field FGF in the CC3DML.** FGF diffuses with diffusion constant 0.1 and is secreted at the rate 2 units per pixel per MCS.

To make cell growth depend on concentration we make small modifications to CellsortingSteppable class:

1. from PySteppables import *
2. import CompuCell
3. import sys
4. class cellsortingSteppable(SteppableBasePy):
5. def __init__(self,_simulator,_frequency=10):
6. SteppableBasePy.__init__(self,_simulator,_frequency)
7. def start(self):
8. for cell in self.cellList:
9. cell.targetVolume=25
10. cell.lambdaVolume=2.0
11. def step(self,mcs):
12. field=CompuCell.getConcentrationField(self.simulator,"FGF")
13. pt=CompuCell.Point3D()
14. for cell in self.cellList:
15. if cell.type==self.CONDENSING:
16. pt.x=int(cell.xCOM)
17. pt.y=int(cell.yCOM)
18. concentration=field.get(pt)
19. cell.targetVolume+=0.01*concentration

**Supplemental List L6**. **Parameterizing cell growth in terms of FGF concentration.**

In line 14 we access reference to the diffusible field. In line 16 we create Point3D object, which is used to access values of the concentration field. We extract center of mass positions in lines 19-20 and convert them to integers so that they can be used to access specific array locations of the concentration field. Once we extract the value of concentration field at the center of mass of Condensing cell we increase target volume by the amount proportional to this concentration line 22.

The results are shown below:


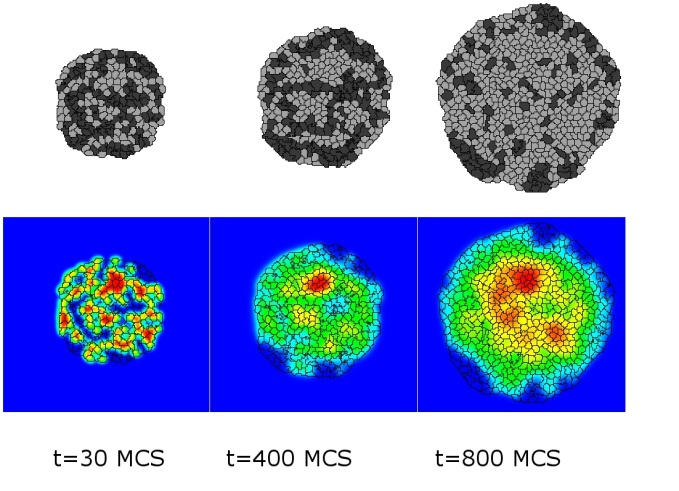
**Supplemental Figure S12.** **Snapshots of the cell-lattice configurations for simulation where target volume of Condensing cells proportional to the concentration of FGF secreted by NonCondensing cells.** Bottom panel shows concentration profile of the FGF. Notice that areas of high FGF concentration overlap with regions occupied by NonCondensing cells.

We have only looked at small subset CC3D capabilities. More complete overview of GGH modeling using CC3D can be found in [1,2,3]. Even though presented examples are relatively simple they do provide a glimpse of rich set of features that CC3D offers to modelers. Almost all published GGH models make extensive use of Python scripting to customize cellular behavior and implement complex intra- and inter-cellular interactions.

# References

1. “Multi-Cell Simulations of Development and Disease Using the CompuCell3D Simulation Environment,” Maciej Swat, Susan D. Hester, Randy W. Heiland, Benjamin L. Zaitlen, James A. Glazier. In Ivan V. Maly ed., *Systems Biology Series: Methods in Molecular Biology*, pp. 138-190.
2. <http://www.compucell3d.org/Manual>
3. “Multi-scale modeling of tissues using CompuCell3D ”, Maciej H. Swat, Gilberto L. Thomas, Julio M. Belmonte, Abbas Shirinifard, Mitja Hmeljak, and James A. Glazier. In Anand R. Asthagiri, Adam Arkin, *Computational Methods in Cell Biology* Volume 110, ISBN: 978-0-12-388403-9.

1. We have graphically edited screenshots of Wizard pages to save space. [↑](#footnote-ref-1)
2. We use indent each nested block by two spaces in all listings in this paper to avoid distracting rollover of text at the end of the line. However, both Simulation Wizard and standard Python use an indentation of four spaces per block. [↑](#footnote-ref-2)
